# Outcome in early vs late intubation among COVID-19 patients with acute respiratory distress syndrome: an updated systematic review and meta-analysis

**DOI:** 10.1038/s41598-022-26234-7

**Published:** 2022-12-14

**Authors:** Denio A. Ridjab, Ignatius Ivan, Fanny Budiman, Dafsah A. Juzar

**Affiliations:** 1 Department of Medical Education Unit, School of Medicine and Health Sciences, Atma Jaya Catholic University of Indonesia, Jakarta, Indonesia; 2 Fifth Year Medical Student, School of Medicine and Health Sciences, Atma Jaya Catholic University of Indonesia, Jakarta, Indonesia; 3 Department of Cardiology and Vascular Medicine, Faculty of Medicine, University of Indonesia, National Cardiovascular Center of Harapan Kita, Jakarta, Indonesia

**Keywords:** Viral infection, Cardiology

## Abstract

Timing of endotracheal intubation in COVID-19 patients with acute respiratory distress syndrome (ARDS) remains controversial regarding its risk and benefit in patient outcomes. Our study aims to elucidate early versus late intubation outcomes among COVID-19 patients with ARDS. A protocol of this study is registered at the international prospective register of systematic reviews (PROSPERO) (CRD42021230272). We report our systematic review based on PRISMA and MOOSE guidelines. We searched the Cochrane Library, EBSCOhost, EMBASE, Grey Literature Report, OpenGrey, ProQuest, PubMed, and ScienceDirect from inception until 4 December 2021. Titles and abstracts were reviewed for their relevance. The risk of bias in each study was evaluated using the risk of bias in non-randomised studies-of interventions (ROBINS-I) guideline. Trial sequential analysis is done to elucidate firm evidence. We retrieved 20 observational studies that assessed an intervention (early vs. late intubation). Meta-analysis for in-hospital mortality reduction showed 119 fewer deaths per 1000 patients in early intubation. Early intubation reduces 2.81 days of ICU length of stay (LOS) and 2.12 days of ventilation duration. Benefits for mortality and ICU LOS reduction were based on studies with low to moderate risk of bias while ventilation duration was based on low disease burden setting. According to the contextualized approach, the benefit of mortality reduction showed a trivial effect, while ICU LOS and ventilation duration showed a small effect. GRADE certainty of evidence for mortality reduction in early intubation is moderate. The certainty of evidence for ICU length of stay, ventilation duration, ventilator-free days, and continuous renal replacement therapy are very low. This updated systematic review provided new evidence that early intubation might provide benefits in treating COVID-19 patients with ARDS. The benefits of early intubation appear to have an important but small effect based on contextualized approach for ICU LOS and ventilation duration. In reducing in-hospital mortality, the early intubation effect was present but only trivial based on contextualized approach. TSA showed that more studies are needed to elucidate firmer evidence.

## Introduction

High mortality from early reports in COVID-19 patients using mechanical ventilators raised concern that respiratory failure due to COVID-19 are different from other cause of ARDS^1–3^. Management of respiratory failure in COVID-19 patients are still being studied whether it should be similar with other ARDS common cause^4^. One of the most important aspect in treating respiratory failure is to decide the timing of intubation, either early or late^4–6^. This is crucial because late mechanical ventilation has been associated with worsened clinical outcome in ARDS^7^. By delaying invasive ventilation, patient may become susceptible to high respiratory drive which lead to self-induced lung injury (SILI)^8^.

A previous meta-analysis has shown that intubation timing may have no influence on mortality and morbidity of COVID-19 patients with ARDS and suggesting that a wait-and-see approach might be justified to reduce the administration of invasive ventilation^9^. There are some limitation that has been pointed out with this report especially regarding the definition of early intubation and other clinical variables that may be taken into consideration which could influence outcome such as respiratory profile and more importantly the severity of illness^10,11^. Other limitation that has been addressed by the authors including the heterogeneity findings, no trial sequential analysis, results that may be affected by confounding factors due to the nature of synthesizing observational studies, and potential bias on the subgroup analysis^9^. On the other hand, several new studies have emerged revealing lower mortality events in favor of early intubation group^12–15^.

In accordance with the consensus on updating systematic review^16^ our aim is to elucidate current evidence on outcome for early versus late intubation among COVID-19 patients with ARDS.

## Methods

### Protocol and registration

We reported our study in accordance with the Preferred Reporting Items for Systematic Reviews and Meta-Analyses (PRISMA) statement and Meta-analysis of Observational Studies in Epidemiology (MOOSE) checklist^17,18^. A protocol of this study was registered at the International Prospective Register of Systematic Reviews (PROSPERO) with registration number: CRD42021230272^19^.

### Data sources

We searched the Cochrane Library, EBSCOhost, EMBASE, ProQuest, PubMed and ScienceDirect from inception until 4 December 2021. Additionaly grey literature databases were also searched using Grey Literature Report and OpenGrey database. Manual searching was also done in Google Scholar.

### Search strategy, study eligibility, and study selection

Using the MeSH terms and [All Field], we complemented search strategy by using combination of keywords as following: COVID-19, respiratory distress syndrome, intubation, timing, mortality, survival, ICU length of stay, ventilation duration, organ-failure free days, adverse events. Similar and relevant terminologies were used to broaden our search strategy.

Studies were reviewed and included based on: (1) population: Adult patients (> 18 years old) with a COVID-19 confirmed diagnosis using reverse-transcriptase polymerase chain reaction (RT-PCR) with ARDS according to Berlin Criteria^20^; (2) intervention: early intubation defined as intubated within 24–48 h of hospital/ICU admission/ARDS diagnosis; (3) comparison: late intubation defined as intubated 24–48 h after hospital/ICU admission/ARDS diagnosis; (4) primary outcome: in-hospital mortality and secondary outcome: ICU length of stay (LOS), ventilation duration, ventilator free days (VFD), continuous renal replacement therapy (CRRT), ICU free days, and adverse events. The type of study included was observational studies that assessed an intervention, in this case regarding early versus late intubation in COVID-19 patients. Case reports or case series were excluded if consisting less than 5 patients. Conference abstracts were included if sufficient information was provided. Studies on non-COVID-19 patients, children (< 18 years old), or pregnant females were excluded.

Two authors independently searched, screened and selected articles using Endnote X9. Disagreement (< 1% of individual assessments) were resolved by discussion with other authors.

### Data collection

Using standardised table, two authors independently extracted data including: authors, publication year, country of origin, sample size, population characteristic (age, sex, commorbidity), eligibility criteria, follow-up duration, intubation timing, and outcome results along with their 95% confidence intervals (CIs), standard deviation (SD), or interquartile ranges (IQRs). We calculated mean and SDs for studies reporting median and IQRs using method as proposed by Wan et al.^21^ Authors of studies were contacted via email to request access for missing data.

### Quality assessment

Two authors independently assessed the risk of bias using the Risk Of Bias In Non-randomised Studies-of Interventions (ROBINS-I) guideline^22^. Certainty of each outcome was evaluated using Grading of Recommendations Assessment, Development and Evaluation (GRADE) system with the GRADEpro Guideline Development Tool^23,24^. Certainty of assessment was based on consideration of risk of bias, inconsistency, indirectness and imprecision. Imprecision was evaluated based on absolute estimates using risk difference (RD) for in-hospital mortality and CRRT while using mean difference (MD) for ICU LOS, ventilation duration and VFD. Disagreement (< 1% of individual assessments) were resolved by discussion with other authors. A contextualized approach was adopted to define minimum benefit thresholds to be able to estimate imprecision^25–27^. Due to no data available on quantitative studies of patient values, the thresholds of small but important effects was chosen by consensus: 1% for in-hospital mortality, 1 day for ICU LOS, 1 day for ventilation duration, 1 day for VFD, and 1% for CRRT^25–27^.

### Meta-analysis

Meta-analysis using RevMan 5.3 was conducted. For dichotomous outcomes, we expressed effect estimates by risk ratios (RR) with 95%CI. For continuous outcomes, MD with standard deviation (SD) was used. We combined means from two different groups in continuous variables based on StatsToDo formula (www.statstodo.com).

In order to detect heterogeneity statistically, we used Cochrane’s Q test (chi-squared test) and Higgins I^2^ statistics. We defined an I^2^ of 25–50% as moderate, 51–75% as substantial, I^2^ > 75% as considerable heterogeneity^28,29^. We performed random effects model (REM) using DerSimonian-Laird method regardless of the statistical heterogeneity because all studies might be conducted with variable clinical practices, patient characteristics, ICU admission criteria and therefore clinical heterogeneity were present^30^. A *p* value less than 0.05 denoted statistical significance.

### Subgroup analysis

Subgroup analysis were performed according to: (1) year of publication (2020 vs. 2021); (2) timing of intubation cut-off (24 h vs. 48 h) which was further be categorized based on timing since: (a) ICU admission, (b) hospital admission or (c) ARDS onset based on available definition from each study; (3) burden of disease in the point of care where the study is conducted (high disease burden) versus (low disease burden). The information regarding the situation of disease burden during the time of each study was collected based on literature reports^9,12,31–35^.

### Sensitivity analysis

We performed sensitivity analysis by performing: (1) leave-one-out meta analysis by excluding a single study to recalculate the pooled effect estimate and finding a study with exaggerated effect size that may distort overal result aiming to achieve I^2^ below 50% or 25% for meta-analysis with I^2^ more than 50 and 25%, respectively^36^; (2) exclusion of studies with serious to critical risk of bias; (3) exclusion of studies that did not report a comparable Sequential Organ Failure Assessment score (SOFA) score between groups; (4) exclusion of studies that did not report a comparable P_a_O_2_/F_I_O_2_ (PF) ratio.

### Publication bias analysis

We used JASP Version 0.14.1 to evaluate publication bias^37^. We generated Begg’s Funnel plot when the number of included studies is at least 10 with no considerable heterogeneity^29,38^. This was further confirmed by additional Egger’s test and Begg and Mazumdar’s rank correlation test^39,40^. Correction of publication bias was based on Duval and Tweedie’s trim and fill method^29,41^.

### Trial sequential analysis

Trial Sequential Analysis (TSA) was performed using Trial Sequential Analysis Software (version 0.9.5.10 Beta; Copenhagen Trial Unit, Copenhagen, Denmark)^42^. Adjustment of Z values threshold were done by using O’Brien–Fleming α-spending function to control the risk of a type-1 error and β-spending function and futility boundaries to control the risk of a type-2 error^42^. A two-sided 95% CI was used and diversity-adjusted estimated information size used α = 0.05 (two-sided) and β = 0.20 (80% power), with an anticipated estimated of: (1) 10 and 20% relative risk reduction (RRR) of mortality, (2) − 2.00 days mean difference in ICU LOS, (3) − 2.00 day mean difference in ventilation duration, (4) 2.00 days mean difference in VFD and (5) 10% and 20% RRR of CRRT, in which all estimation were model-variance based heterogeneity correction. The variance was calculated from data obtained from the trials included in this meta-analysis.

## Results

**Figure 1:**
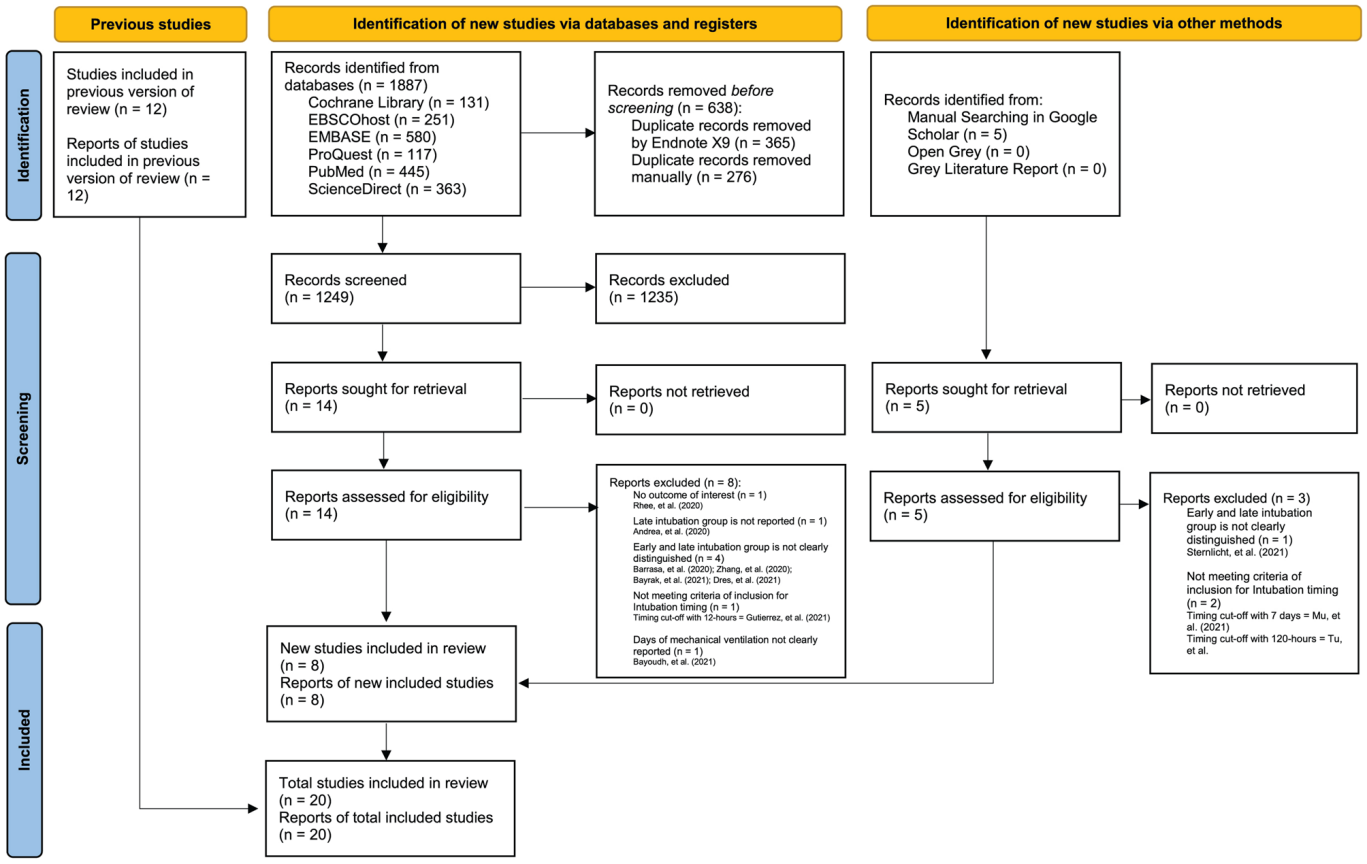
PRISMA 2020 Flow Diagram. Studies selection process are shown in this flow diagram.

Our search strategy identified a total of 1887 studies (Supplementary Tables S1–S3). Results were imported into Endnote X9, and duplicates removed, leaving 1249 articles to be reviewed. We also collected results from manual searching in which 5 articles were retrieved. Abstracts were reviewed for relevance based on inclusion and exclusion criteria. After screening, 14 articles from database searching and 5 articles from manual searching were retained for full-review. We excluded 8 studies from database searching results and 3 studies from manual searching result with reasons that can be found in Fig. 1, leaving new 8 studies^12–15,43–46^ for updating previous systematic review^9^. We complemented 12 studies^4,31,32,47–55^ from previous systematic review^9^ for our final search results and thus a total of 20 studies were evaluated for this systematic review (See Fig. 1 for details of this process). All 20 studies (Supplementary Table S4) reported number of in-hospital mortality. There were 10 studies^4,12,13,15,31,43,46,51,52,54^ reported ICU LOS, 10 studies^4,12,13,15,31,32,46,51,52,54^ reported ventilation duration, 4 studies^32,43,46,51^ reported VFD, and 4 studies^12,32,46,52^ reported adverse events related to the use of continuous renal replacement therapy (CRRT).

Sixteen studies^4,12–15,31,32,44,45,47,48,50–52,54,55^ included are retrospective cohort, and four studies^43,46,49,53^ are prospective cohort. Country of origin of seven studies^4,12–14,45,52,54^ are United States, two studies^31,55^ are from Germany, two studies^48,50^ are from Italy, and each of one study is from South Korea^51^, India^15^, Greece^32^, Portugal^44^, France^43^, Chile^46^, Tunisia^47^, and there is one multinational study^53^ from Spain and Andorra, and another^49^ from France, Switzerland and Belgium. This systematic review included a total of 12,139 patients with age range from 30 to 89 years old. All patients included was hospitalized with SARS-CoV2 infection confirmed by using RT-PCR and diagnosed with ARDS in accordance with Berlin criteria. The definition of early and late intubation between studies were based on intubation timing cut-off 24 hours^4,12,13,31,32,44,47–51,53,55^ or 48 h^14,15,43,45,46,52^. Study by Pandya et al.^54^ have a slightly different categorization with 1.27 days for time distinction. Supplementary Table S4 summarize characteristic of included studies.

## Primary outcome

### In-hospital mortality

We evaluated all 20 studies for in-hospital mortality risk in COVID-19 patients with ARDS who were intubated early or late. In all 20 studies pooled together, there was no significant difference for in-hospital mortality between group (44.25% vs. 41.98%; RR 0.95, 95%CI 0.86–1.05, *p* = 0.29; I^2^ = 53%) (Supplementary Fig. S1).

### Subgroup analysis for in-hospital mortality

**Figure 2:**
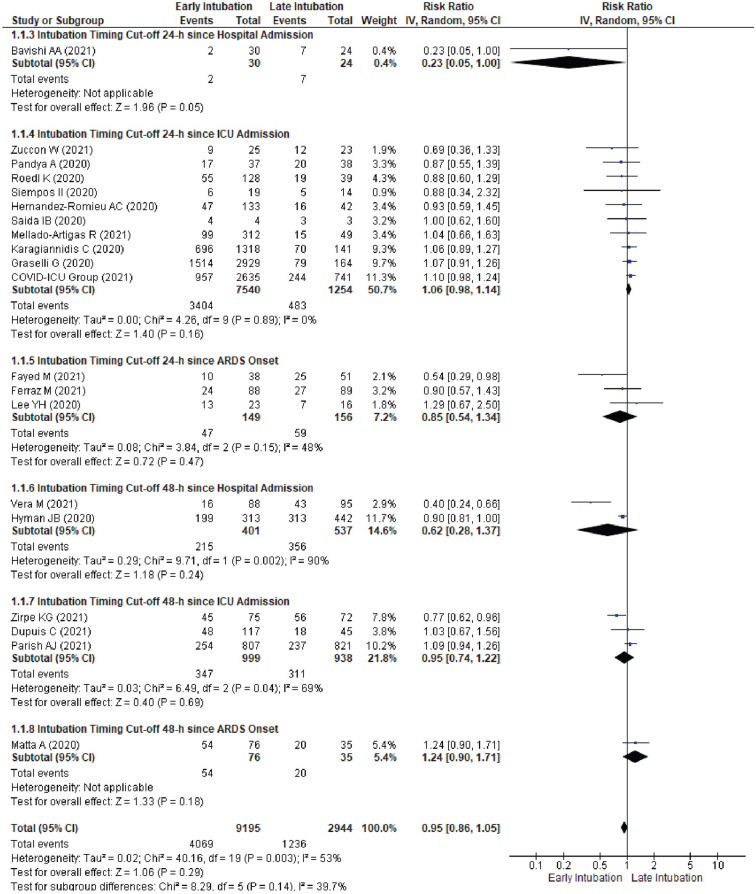
Subgroup Analysis According to Intubation Timing Categorized based on Timing since Hospital Admission, ICU Admission, or Acute Respiratory Distress Syndrome Onset for Risk of ICU-Mortality between Early Intubation Compared with Late Intubation Group. The solid squares denote the risk ratio, with the horizontal lines indicating the 95% confidence intervals and the diamond denotes the pooled effect size. ARDS, acute respiratory distress syndrome; Chi^2^ , chi-squared statistic; CI , confidence interval; df , degrees of freedom; I^2^ , I-squared heterogeneity statistic; ICU, intensive-care unit; IV , inverse variance; p , *p* value; SD , standard deviation; Z , Z statistic.

Subgroup analysis based on publication year in 2020 and 2021 (Supplementary Fig. S2); intubation timing with 24-h and 48-h cut-off (Supplementary Fig. S3); high and low disease burden setting (Supplementary Fig. S4) showed a comparable in-hospital mortality between groups. Pooled analysis based on intubation timing within 24-h since hospital or ICU admission or ARDS onset, and 48-h since hospital or ICU admission or ARDS onset also showed comparable in-hospital mortality between groups (Fig. 2).

### Sensitivity analysis for in-hospital mortality

**Figure 3:**
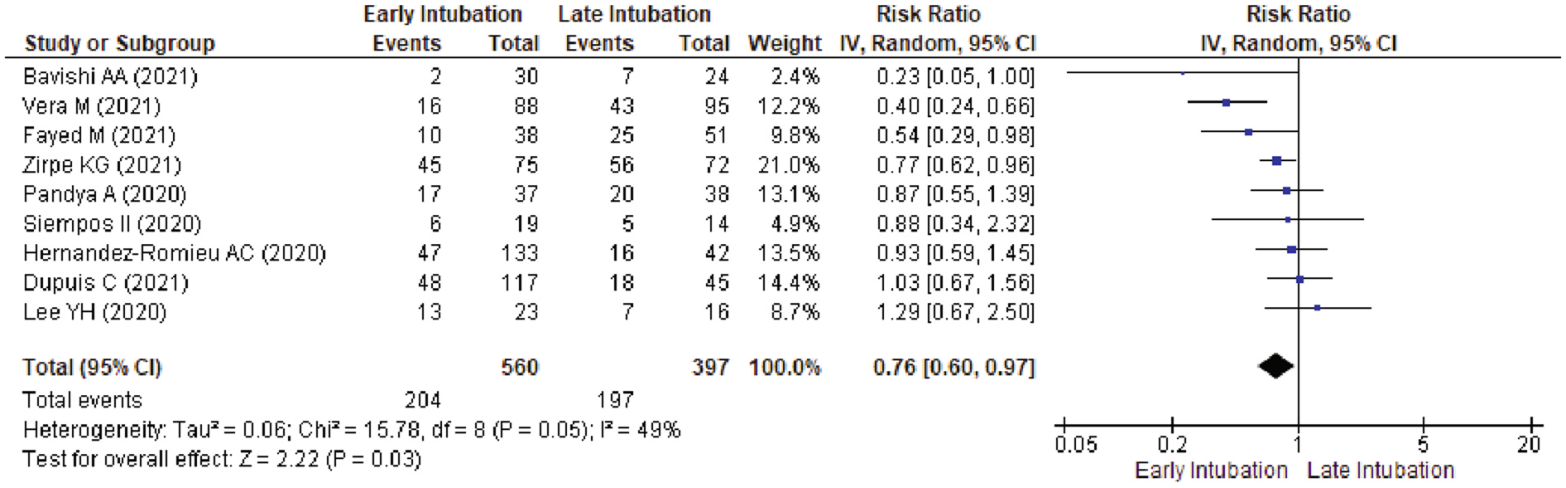
Sensitivity Analysis by Excluding Studies with Serious to Critical Risk of Bias for Risk of ICU-Mortality between Early Intubation Compared with Late Intubation Group. The solid squares denote the risk ratio, with the horizontal lines indicating the 95% confidence intervals and the diamond denotes the pooled effect size. Chi^2^ , chi-squared statistic; CI , confidence interval; df , degrees of freedom; I^2^ , I-squared heterogeneity statistic; IV , inverse variance; p , *p* value; SD , standard deviation; Z , Z statistic.

Leave-one-out analysis showed no change for in-hospital mortality risk for pooled meta-analysis of all 20 studies (Supplementary Table S5), subgroup of publication year (Supplementary Table S6), intubation timing (Supplementary Table S7), and disease burden (Supplementary Table S8). Meta-analysis in studies with low to moderate risk of bias showed a significantly lower in-hospital mortality rate in early intubation group (36.43% vs. 49.62%; RR 0.76, 95%CI 0.60–0.97, *p* = 0.03; I^2^ = 49%) (Fig. 3). A further leave-one-out analysis excluding Vera et al.^46^ was performed and showed a borderline significant risk reduction (RR 0.83, 95%CI, 0.69–1.00; *p* = 0.05; I^2^ = 16%) (Supplementary Table S9). Meta-analysis in studies reporting a comparable SOFA score between groups showed no significant difference for in-hospital mortality rate between groups (Supplementary Fig. S5). A further leave-one-out analysis excluding Lee et al.^51^ change the result and showed a significantly lower risk of in-hospital mortality for early intubation group (38.89% vs. 57.76%; RR, 0.69, 95%CI 0.50–0.94, *p* = 0.02; I^2^ = 20%) (Supplementary Table S10). Meta-analysis done in studies reporting a comparable P_a_O_2_/F_I_O_2_ ratio between groups showed no significant difference (Supplementary Fig. S6). A further leave-one-out analysis excluding Vera et al. showed a similar result (Supplementary Table S11).

### Trial sequential analysis for in-hospital mortality

All TSA on in-hospital mortality with an estimated risk reduction of 10% and 20% can be found in Supplementary Figs. S7–S30. TSA showed that more evidences are still needed to achieve a conclusive findings for analysis based on intubation timing cut-off 24-h since ARDS onset, intubation timing cut-off 48-h since hospital and ICU admission, low disease burden setting (for 10% RRR), studies with low to moderate risk of bias, studies reporting comparable SOFA or P_a_O_2_/F_I_O_2_ ratio between groups (for 10% RRR).

## Secondary outcome

### ICU length of stay

We evaluated 10 studies for ICU LOS in COVID-19 patients with ARDS who were intubated early or late. In all 10 studies pooled together, there was no significant difference for ICU LOS between early and late intubation group (MD -2.24, 95%CI − 4.55–0.07, *p* = 0.06; I^2^ = 72%) (Supplementary Fig. S31).

### Subgroup analysis for ICU length of stay

Subgroup analysis based on publication year in 2020 and 2021 (Supplementary Fig. S32), intubation timing with 48-h cut-off (Supplementary Fig. S33), high disease burden setting (Supplementary Fig. S34) showed a comparable ICU LOS between group. Analysis based on intubation timing with 24 h cut-off showed a reduced ICU LOS for early intubation group (MD − 2.85, 95%CI − 5.64 to − 0.05, *p* = 0.05, I^2^ = 61%) (Supplementary Fig. S33). Analysis for studies done in low disease burden showed a reduced ICU LOS for early intubation group (MD − 5.64, 95%CI − 9.70 to − 1.58, *p* = 0.007, I^2^ = 72%) (Supplementary Fig. S34).

### Sensitivity analysis for ICU length of stay

**Figure 4:**
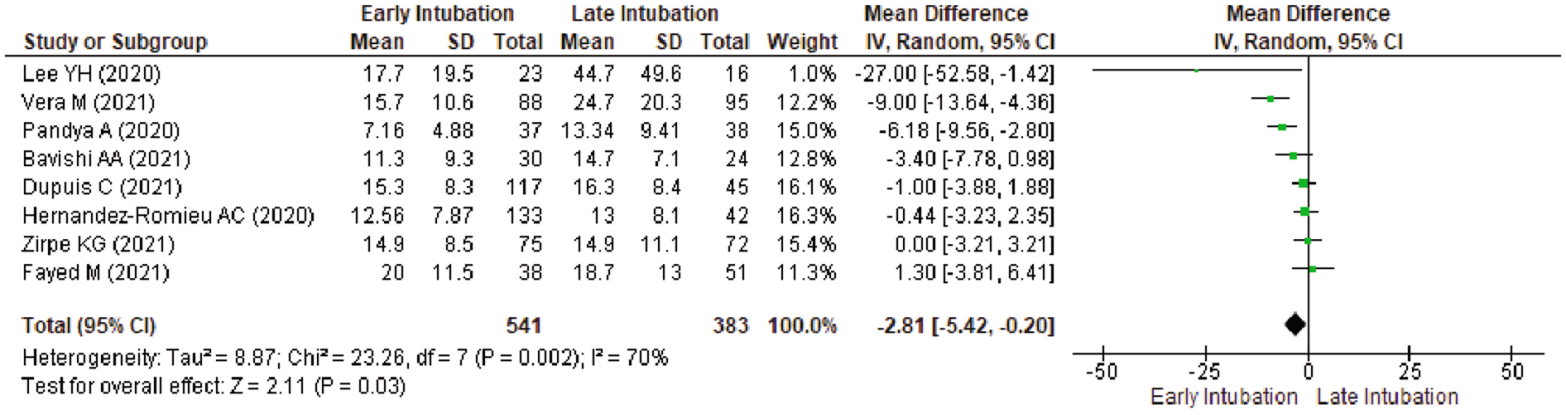
Sensitivity Analysis by Excluding Studies with Serious to Critical Risk of Bias for ICU Length of Stay between Early Intubation Compared with Late Intubation Group. The solid squares denote the mean difference, with the horizontal lines indicating the 95% confidence intervals and the diamond denotes the pooled effect size. Chi^2^ , chi-squared statistic; CI , confidence interval; df , degrees of freedom; I^2^ , I-squared heterogeneity statistic; IV , inverse variance; p , *p* value; SD , standard deviation; Z , Z statistic.

Leave-one-out analysis showed no change in ICU LOS for pooled meta-analysis of all 10 studies (Supplementary Table S12), subgroup of publication year (Supplementary Table S13), and high disease burden setting (Supplementary Table S14). When performed in subgroup of intubation timing (Supplementary Table S15) and low disease burden (Supplementary Table S14), results are change to not significant after exclusion of study with the most exaggerated effect. Meta-analysis done in studies with low to moderate risk of bias showed a reduced ICU LOS in early intubation group (MD − 2.81, 95%CI − 5.42 to − 0.20, *p* = 0.03; I^2^ = 70%) (Fig. 4). A further leave-one-out analysis excluding Vera et al.^46^ was performed and change result to not significant (Supplementary Table S16). Meta-analysis in studies reporting a comparable SOFA score between groups showed no significant difference (Supplementary Fig. S35). A further leave-one-out analysis excluding Lee et al.^51^ showed a comparable result (Supplementary Table S17). Meta-analysis in studies reporting a comparable P_a_O_2_/F_I_O_2_ ratio between groups showed no significant difference (Supplementary Fig. S36). A further leave-one-out analysis excluding Vera et al.^46^ showed a comparable result (Supplementary Table S18).

### Trial sequential analysis for ICU length of stay

All TSA on ICU LOS with an estimated mean difference reduction of − 2.00 days can be found in Supplementary Figs. S37–S44. TSA showed that more evidences are still needed to achieve a conclusive findings for analysis based on intubation timing cut-off 24-h and 48-h, low disease burden setting, studies with low to moderate risk of bias, studies reporting comparable SOFA score between groups and comparable P_a_O_2_/F_I_O_2_ ratio between groups.

### Ventilation duration

We evaluated 10 studies for ventilation duration in COVID-19 patients with ARDS who were intubated early or late. In all 10 studies pooled together, there was no significant difference for ventilation duration between early and late intubation group (MD − 0.33, 95%CI − 2.14–1.49, *p* = 0.73). Moderate heterogeneity was detected (I^2^ = 59%) (Supplementary Fig. S45).

### Subgroup analysis for ventilation duration

**Figure 5:**
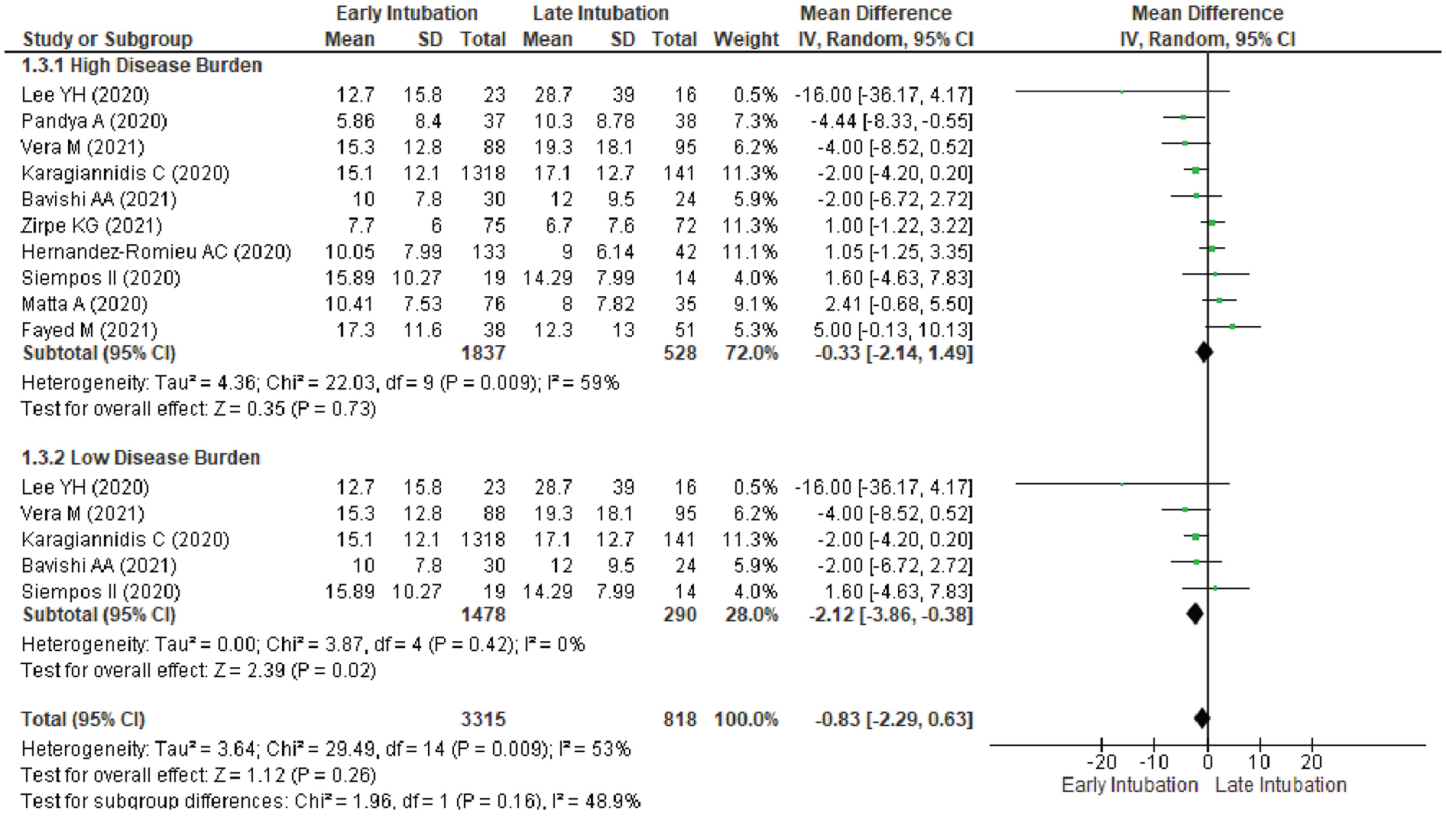
Subgroup Analysis According to Disease Burden for Ventilation Duration between Early Intubation Compared with Late Intubation Group. The solid squares denote the mean difference, with the horizontal lines indicating the 95% confidence intervals and the diamond denotes the pooled effect size. Chi^2^ , chi-squared statistic; CI , confidence interval; df , degrees of freedom; I^2^ , I-squared heterogeneity statistic; IV , inverse variance; p , *p* value; SD , standard deviation; Z , Z statistic.

Subgroup analysis based on publication year in 2020 and 2021 (Supplementary Fig. S46); intubation timing with 24-h and 48-h cut-off (Supplementary Fig. S47); and high disease burden setting (Fig. 5) showed a comparable ventilation duration between group. For studies done in low disease burden showed a reduced ventilation duration for early intubation (MD − 2.12, 95%CI − 3.86 to − 0.38, *p* = 0.02, I^2^ = 0%) (Fig. 5).

### Sensitivity analysis for ventilation duration

Leave-one-out analysis showed no change for in-hospital mortality risk for pooled meta-analysis of all 10 studies (Supplementary Table S19), subgroup of publication year (Supplementary Table S20), and intubation timing (Supplementary Table S21). When performed in studies with high disease burden setting, result for ventilation duration change and become significant after exclusion of study by Pandya et al.^54^ with increase ventilation duration for early intubation versus late intubation group (MD 1.58, 95%CI 0.21–2.95, *p* = 0.02; I^2^ = 0%) (Supplementary Table S22). Meta-analysis in studies with low to moderate risk of bias showed no different ventilation duration between groups (Supplementary Fig. S48). A further leave-one-out analysis excluding Pandya et al.^54^ was performed and showed no significant difference between groups (Supplementary Table S23). Meta-analysis in studies reporting a comparable SOFA score between groups also showed no significant difference (Supplementary Fig. S49). A further leave-one-out analysis excluding Lee et al.^51^ showed a similar result (Supplementary Table S24). Meta-analysis in studies reporting a comparable P_a_O_2_/F_I_O_2_ ratio between groups showed no significant difference (Supplementary Fig. S50). A further leave-one-out analysis excluding Pandya et al.^54^ showed a similar result (Supplementary Table S25).

### Trial sequential analysis for ventilation duration

All TSA on ventilation duration with an estimated mean difference reduction of − 2.00 days can be found in Supplementary Figs. S51–S58. TSA showed a firm evidence that there was an increase ventilation duration for early intubation for studies with high disease burden setting. TSA showed that more evidences are still needed to achieve a conclusive findings for analysis based on intubation timing cut-off 24-h and 48-h, low disease burden setting, studies with low to moderate risk of bias, studies reporting comparable SOFA score between groups.

### Ventilator-free days

We evaluated 4 studies for VFD in COVID-19 patients with ARDS who were intubated early or late. In all studies pooled together, there was no significant difference between group (MD − 0.84, 95%CI − 4.80–3.12, *p* = 0.68; I^2^ = 75%) (Supplementary Fig. S59). Subgroup analysis is not performed due to limited number of studies. Leave-one-out analysis showed no change in VFD for pooled meta-analysis of all 4 studies (Supplementary Table S26). TSA showed that more studies are necessary to confirm evidence (Supplementary Fig. S60).

### Continuous renal replacement therapy

We evaluated 4 studies for CRRT in COVID-19 patients with ARDS who were intubated early or late. In all 4 studies pooled together, there was no significant difference for CRRT between early and late intubation group (MD 0.65, 95%CI 0.27–1.55, *p* = 0.33; I^2^ = 75%) (Supplementary Fig. S61). Leave-one-out analysis showed stable results (Supplementary Table S27). TSA showed that more studies are necessary to confirm the evidence (Supplementary Fig. S62–S63).

### ICU free days

ICU-free days was only reported by Siempos et al.^32^ and result showed that there was no significant difference between early and late intubation with a median of 0 day (0–16 days) for early intubation and 0 day (0–4 days) for late intubation. Currently, there has been no study evaluate organ-failure free days between early and late intubation.

### Other adverse events

Based on Lee et al.^51^ number of events for acute kidney injury, acute cardiac injury, catheter–related blood stream infection, bleeding, cardiopulmonary–cerebral resuscitation was not different between early and late intubation. Lee et al.^51^ and Siempos et al.^32^ did not find a significant difference on number of events for septic shock between early and late intubation. Lee et al.^51^ and Ferraz et al.^44^ did not find a significant difference on number of events for ventilator associated pneumonia.

### Quality assessment, quality of evidence and publication bias

Quality assessment using ROBINS-I shows all studies’ risk of bias result range from moderate to critical. Complete reasoning can be found in Supplementary Table S28. GRADE certainty of evidences are judged to be moderate for mortality reduction and very low for ICU LOS, ventilation duration, VFD, and CRRT (Supplementary Table S29). Additionaly, Supplementary Figs. S64–S68 showed the minimum benefit threshold of in-hospital mortality, ICU LOS, ventilation duration, VFD, and CRRT between early and late intubation that used to evaluate imprecision. We did not downgrade the level of evidence for imprecision of in-hospital mortality because the pooled effect is between the minimum benefit threshold and null effect showing that early intubation has a trivial effect in reducing mortality [Supplementary Fig. S64]. We downgraded one level of evidence for imprecision of ICU LOS because the pooled effect crossed the minimum benefit threshold but the upper confidence interval reached the area of trivial effect. Thus, early intubation probably reduces ICU LOS [Supplementary Fig. S65]. We downgraded one level of evidence for imprecision of ventilation duration because the pooled effect crossed the minimum benefit threshold but the upper confidence interval fell on the area of trivial effect. Thus, early intubation probably reduces ventilation duration [Supplementary Fig. S66]. We downgraded two level of evidence for imprecision of VFD because the point estimate of pooled effect is on the area of trivial effect while the confidence interval crossed the minimum benefit and harm threshold. Thus, early intubation may have trivial or no effect in VFD [Supplementary Fig. S67]. We did not downgrade the level of evidence for imprecision of CRRT because the pooled effect is between the minimum benefit and harm threshold. Thus, early intubation has a trivial or no effect for risk in using CRRT [Supplementary Fig. S68].

A funnel plot with trim and fill analysis is generated for in-hospital mortality (Supplementary Fig. S69), ICU LOS (Supplementary Fig. S70), and ventilation duration (Supplementary Fig. S71). Egger’s test showed no publication bias for in-hospital mortality (Z = − 1.679, *p* = 0.093) and ventilation duration (Z = − 1.130, *p* = 0.259) but significant result was found in ICU LOS (Z = − 2.167, *p* = 0.030). Rank correlation test showed no publication bias for in-hospital mortality (Tau = − 0.274, *p* = 0.098), ICU LOS (Tau = − 0.289, *p* = 0.291), and ventilation duration (Tau = 0.067, *p* = 0.862). Overall, a minor publication bias may exist.

## Discussion

We found that early intubation is related to 24% relative risk reduction of in-hospital mortality when adjusted with only studies with low to moderate risk of bias. However, the result become borderline significant after a leave-one-out analysis was performed. On the other hand, our result also suggests a 31% lower risk of in-hospital mortality after leave-one-out analysis was performed in studies reporting comparable SOFA score. Although SOFA adjustment method showed a lower heterogeneity (I^2^ = 20% vs. I^2^ = 49%), the number of included studies are however smaller (4 studies vs. 9 studies) when compared with analysis based on study quality. TSA showed that more studies are needed to make a firm evidence for both analysis. In early intubation, there are 119 fewer death per 1000 patients based on studies with low to moderate risk of bias. According to the contextualized approach, this effect is considered to be trivial.

In our analysis, early intubation was also related to 2.85 days (6 studies) or 5.64 days (4 studies) reduction of ICU LOS based on intubation timing cut-off 24 h or when studies done in low disease burden setting, respectively. However, both results reveal substantial heterogeneity and a leave-one-out analysis performed on both adjustments showed unstable result. Studies with low to moderate risk of bias showed a 2.81 days reduction of ICU LOS. Although the number of studies is larger (8 studies), heterogeneity is still substantial while a leave-one-out analysis showed unstable result. According to the contextualized approach, this effect is considered to be small.

Our analysis in ventilation duration showed an interesting result revealing 1.58 days increase of ventilation duration for early intubation in high disease burden setting. In early intubation, there are 2.12 days reduction of ventilation duration based on studies in low disease burden setting. According to the contextualized approach, this effect is considered to be small. Our analysis showed no benefit or harm of VFD, CRRT, ICU-free days or other adverse events between groups. Based on analysis, there is a minor publication bias for in-hospital mortality, ICU-LOS and ventilation duration outcome.

We addressed some important limitations provided by previous systematic review^9^ and other reports^10,11^. We found that clinical parameters (SOFA score), level of disease burden, and study quality are important to be adjusted for elucidating benefit or harm of intubation timing. We also included TSA in our analysis to control risk of type-1 and type-2 error^42^. All positive findings supporting early intubation still required more studies for a conclusive meta-analysis. Our TSA show firm evidence for increase ventilation duration when early intubation done in high disease burden setting. By adding up 8 new studies^12–15,43–46^ and modifying subgroup and sensitivity analysis, current meta-analysis revealed different results compared to the previous study^9^ but in favor of prior international guidelines^56–59^. We did not perform sensitivity analysis based on a prior trial of high flow nasal cannula or non-invasive ventilation because previous report suggested that this additional definition might classify the same subject into either early or late intubation group and thereby have a vastly different implication^10^.

Previous recommendation from experts suggested that early intubation and invasive ventilation is in favor to prevent too vigorous patient’s respiratory efforts that may trigger SILI and affect short- and long-term clinical outcome^6^. Work of breathing remain to be one of the decision basis in judging whether to intubate early or late^6^. Factors such as tidal volume, respiratory rate, minute ventilation, and worsening respiratory status may indicate early intubation^6,60^. Additionally, more objective measurement to indicate intubation timing can be calculated in pneumonia patients with ARDS by using ratio of oxygen saturation (ROX) index^61^. One study^62^ validate this tool based on admission day. A ROX less than 25.26, 21.34 or 11.71 at day 1, 2 or 3 of admission, respectively, are significantly associated with intubation^62^. Currently, there are only 2 studies^13,53^ included in our analysis which report ROX index between groups and thus, further pooled analysis is limited. Future studies may evaluate outcomes by incorporating variables in work of breathing along with ROX index to elucidate risk and benefit for early intubation.

Our systematic review has limitations because all studies are observational. GRADE certainty of evidence is currently very low for early intubation benefit in ICU LOS, ventilation duration, VFD, and CRRT. Due to the nature of observational study, subjective factors including clinician, patient and family preferences along with ventilator availability, institutional culture, and the fact that more severe patients might get earlier intubation, were unable to be controlled by the authors. However, our analysis has included a subgroup based on study quality. A prospective, randomized trials with a large sample would be crucial in the future to address unmeasured confounders and confirmed the results. Other limitation is that our analysis for VFD and CRRT have considerable heterogeneity and this might be due to heterogeneity from population characteristics and clinical practices.

## Conclusion

This updated systematic review provided new evidence that early intubation might provide benefits in treating COVID-19 patients with ARDS. The benefits of early intubation appear to have an important but small effect based on contextualized approach with 2.81 days shorter of ICU LOS, and 2.12 days shorter of ventilation duration. In reducing in-hospital mortality, the early intubation effect was present but only trivial based on contextualized approach with 119 fewer deaths per 1000 patients. TSA showed that more studies are needed to elucidate firmer evidence.

## Supplementary Information

Supplementary Information 1.

Supplementary Information 2.

Supplementary Information 3.

Supplementary Information 4.

Supplementary Information 5.

## Data Availability

All data generated or analysed during this study are included in this published article [and its supplementary information files].

## Abbreviations

APACHE: Acute physiology and chronic health evaluation
ARDS: Acute respiratory distress syndrome
CI: Confidence interval risk ratios (RR)
COVID-19: Coronavirus disease 2019
CRRT: Continuous renal replacement therapy
FEM: Fixed effect model
GCS: Glasgow coma scale
MD: Mean difference
PRISMA: Preferred reporting items for systematic reviews and meta-analyses
PROSPERO: Prospective register of systematic reviews
RT-PCR: Reverse-transcriptase polymerase chain reaction
HR: Hazard ratios
LOS: Length of stay
MV: Mechanical ventilator
OR: Odd ratios
PF: P_a_O_2_:F_I_O_2_
REM: Random effect model
RR: Risk ratios
RRR: Relative risk reduction
SILI: Self-induced lung injury
SOFA: Sequential organ failure assessment
TSA: Trial sequential analysis
VFD: Ventilator free days
