# Supplementary material for: Outcome in early vs late intubation among COVID-19 patients with acute respiratory distress syndrome: an updated systematic review and meta-analysis
Source: Sci Rep. 2022 Dec 14;12:21588. doi: 10.1038/s41598-022-26234-7 (PMC9748395; doi:10.1038/s41598-022-26234-7)
Supplement: Supplementary file 3 — Supplementary Information 3. [file 41598_2022_26234_MOESM3_ESM.docx]

**Supplementary Table S1-S3, S5-S28**

**Manuscript Title:** Outcome in Early vs Late Intubation among COVID-19 Patients with Acute Respiratory Distress Syndrome: An Updated Systematic Review and Meta-Analysis

**Author List:** Denio A. Ridjab^1^, Ignatius Ivan^2^, Fanny Budiman^2^, Dafsah A. Juzar^3^

^1^Department of Medical Education Unit, School of Medicine and Health Sciences, Atma Jaya Catholic University of Indonesia, Jakarta, Indonesia

^2^School of Medicine and Health Sciences, Atma Jaya Catholic University of Indonesia, Jakarta, Indonesia

^3^Department of Cardiology and Vascular Medicine, Faculty of Medicine, Universitas Indonesia/Harapan Kita National Cardiovascular Centre

**Supplementary Table S1.** Search Strategy in Journal Databases

| **Search Terms** | **Database** | **Total** |
| --- | --- | --- |
| (COVID-19[MeSH Terms] OR "COVID-19"[All Fields] OR "Coronavirus Disease-19"[All Fields] OR "2019-nCoV"[All Fields] OR "2019 Novel Coronavirus"[All Fields] OR "Coronavirus Disease 2019"[All Fields] OR "SARS-CoV-2"[All Fields] OR "SARS Coronavirus 2"[All Fields]) AND ("Respiratory Distress Syndrome"[MeSH Terms] OR "Respiratory Insufficiency"[MeSH Terms] OR "Respiratory Distress Syndrome"[All Fields] OR "Respiratory Insufficiency"[All Fields] OR "Respiratory Depression"[All Fields] OR "Acute Respiratory Distress Syndrome"[All Fields] OR "Acute Hypoxemic Respiratory Failure"[All Fields]) AND ("intubation, intratracheal"[MeSH Terms] OR "respiration, artificial"[MeSH Terms] OR "Endotracheal Intubation"[All Fields] OR "Mechanical Ventilation"[All Fields] OR "Invasive Ventilation"[All Fields] OR "Artificial Respiration"[All Fields] OR "Intubation"[All Fields]) AND ("Early"[All Fields] OR "Late"[All Fields] OR "delay"[All Fields] OR "delayed"[All Fields] OR "delaying"[All Fields] OR "delays"[All Fields] OR "Timely"[All Fields] OR "timing"[All Fields] OR "timings"[All Fields] OR "time"[MeSH Terms] OR "time"[All Fields] OR "Prompt"[All Fields] OR "Rapid"[All Fields] OR "Quick"[All Fields] OR "Timely"[All Fields] OR "Fast"[All Fields] OR "Slow"[All Fields]) AND (mortality[MeSH Terms] OR "survival"[MeSH Terms] OR "mortality"[MeSH Terms] OR "mortality"[All Fields] OR "mortalities"[All Fields] OR "mortality"[MeSH Subheading] OR "mortality"[MeSH Subheading] OR "mortality"[All Fields] OR "survival"[All Fields] OR "survival"[MeSH Terms] OR "survivability"[All Fields] OR "survivable"[All Fields] OR "survivals"[All Fields] OR "survive"[All Fields] OR "survived"[All Fields] OR "survives"[All Fields] OR "surviving"[All Fields] OR "death"[MeSH Terms] OR "death"[All Fields] OR "deaths"[All Fields] OR "ICU-Free Days"[All Fields] OR "Ventilator-Free Days"[All Fields] OR "Organ Failure-Free Days"[All Fields] OR "Adverse Events"[All Fields] OR "Adverse Outcomes"[All Fields] OR "Ventilation Duration"[All Fields] OR "ICU Length of Stay"[All Fields]) | Pubmed | 262 |
| ( TI ( “COVID-19” OR “Coronavirus Disease-19” OR "2019-nCoV" OR "2019 Novel Coronavirus" OR "Coronavirus Disease 2019" OR "SARS-CoV-2" OR "SARS Coronavirus 2" ) OR AB ( “COVID-19” OR “Coronavirus Disease-19” OR "2019-nCoV" OR "2019 Novel Coronavirus" OR "Coronavirus Disease 2019" OR "SARS-CoV-2" OR "SARS Coronavirus 2" ) ) AND ( TI ( “Respiratory Distress Syndrome” OR "Respiratory Insufficiency" OR "Respiratory Depression" OR "Acute Hypoxemic Respiratory Failure" ) OR AB ( “Respiratory Distress Syndrome” OR "Respiratory Insufficiency" OR "Respiratory Depression" OR "Acute Hypoxemic Respiratory Failure" ) ) AND ( TI ( "Endotracheal Intubation" OR "Mechanical Ventilation" OR "Invasive Ventilation" OR "Artificial Respiration" OR "Intubation" ) OR AB ( "Endotracheal Intubation" OR "Mechanical Ventilation" OR "Invasive Ventilation" OR "Artificial Respiration" OR "Intubation" ) ) AND ( TI ( "Early" OR "Late" OR "delay" OR "delayed" OR "delaying" OR "delays" OR "Timely" OR "timing" OR "timings" OR "time" OR "time" OR "Prompt" OR "Rapid" OR "Quick" OR "Timely" OR "Fast" OR "Slow" ) OR AB ( "Early" OR "Late" OR "delay" OR "delayed" OR "delaying" OR "delays" OR "Timely" OR "timing" OR "timings" OR "time" OR "time" OR "Prompt" OR "Rapid" OR "Quick" OR "Timely" OR "Fast" OR "Slow" ) ) AND ( TI ( “Mortality” OR “Mortalities” OR “Survival” OR “Survivals” OR “Survived” OR “Survives” OR “Surviving” OR “Surviveable” OR “Survivability” OR “Death” OR “Deaths” OR "ICU-Free Days" OR "Ventilator-Free Days" OR "Organ Failure-Free Days" OR "Adverse Events" OR "Adverse Outcomes" OR "Ventilation Duration" OR "ICU Length of Stay" ) OR AB ( “Mortality” OR “Mortalities” OR “Survival” OR “Survivals” OR “Survived” OR “Survives” OR “Surviving” OR “Surviveable” OR “Survivability” OR “Death” OR “Deaths” OR "ICU-Free Days" OR "Ventilator-Free Days" OR "Organ Failure-Free Days" OR "Adverse Events" OR "Adverse Outcomes" OR "Ventilation Duration" OR "ICU Length of Stay" ) ) | EBSCOHOST | 147 |
| (ti(“COVID-19”) OR ti(“Coronavirus Disease-19”) OR ti("2019-nCoV") OR ti("2019 Novel Coronavirus") OR ti("Coronavirus Disease 2019") OR ti("SARS-CoV-2") OR ti("SARS Coronavirus 2") OR ab(“COVID-19”) OR ab(“Coronavirus Disease-19”) OR ab("2019-nCoV") OR ab("2019 Novel Coronavirus") OR ab("Coronavirus Disease 2019") OR ab("SARS-CoV-2") OR ab("SARS Coronavirus 2")) AND (ti(“Respiratory Distress Syndrome”) OR ti("Respiratory Insufficiency") OR ti("Respiratory Depression") OR ti("Acute Hypoxemic Respiratory Failure") OR ab(“Respiratory Distress Syndrome”) OR ab("Respiratory Insufficiency") OR ab("Respiratory Depression") OR ab("Acute Hypoxemic Respiratory Failure")) AND (ti("Endotracheal Intubation") OR ti("Mechanical Ventilation") OR ti("Invasive Ventilation") OR ti("Artificial Respiration") OR ti("Intubation") OR ab("Endotracheal Intubation") OR ab("Mechanical Ventilation") OR ab("Invasive Ventilation") OR ab("Artificial Respiration") OR ab(“Intubation”)) AND (ti("Early") OR ti("Late") OR ti("delay") OR ti("delayed") OR ti("delaying") OR ti("delays") OR ti("Timely") OR ti("timing") OR ti("timings") OR ti("time") OR ti("time") OR ti("Prompt") OR ti("Rapid") OR ti("Quick") OR ti("Timely") OR ti("Fast") OR ti("Slow") OR ab("Early") OR ab("Late") OR ab("delay") OR ab("delayed") OR ab("delaying") OR ab("delays") OR ab("Timely") OR ab("timing") OR ab("timings") OR ab("time") OR ab("time") OR ab("Prompt") OR ab("Rapid") OR ab("Quick") OR ab("Timely") OR ab("Fast") OR ab("Slow")) AND (ti(“Mortality”) OR ti(“Mortalities”) OR ti(“Survival”) OR ti(“Survivals”) OR ti(“Survived”) OR ti(“Survives”) OR ti(“Surviving”) OR ti(“Surviveable”) OR ti(“Survivability”) OR ti(“Death”) OR ti(“Deaths”) OR ti("ICU-Free Days") OR ti("Ventilator-Free Days") OR ti("Organ Failure-Free Days") OR ti("Adverse Events") OR ti("Adverse Outcomes") OR ti("Ventilation Duration") OR ti("ICU Length of Stay") OR ab(“Mortality”) OR ab(“Mortalities”) OR ab(“Survival”) OR ab(“Survivals”) OR ab(“Survived”) OR ab(“Survives”) OR ab(“Surviving”) OR ab(“Surviveable”) OR ab(“Survivability”) OR ab(“Death”) OR ab(“Deaths”) OR ab("ICU-Free Days") OR ab("Ventilator-Free Days") OR ab("Organ Failure-Free Days") OR ab("Adverse Events") OR ab("Adverse Outcomes") OR ab("Ventilation Duration") OR ab("ICU Length of Stay")) | PROQUEST | 65 |
| “COVID-19” AND “Respiratory Distress Syndrome” AND “Intubation" AND (Timing OR Early OR Delayed) AND (“Mortality” OR “Survival”) | Science Direct | 805 |
| “COVID-19” AND “Respiratory Distress Syndrome” AND “Intubation" AND (Timing OR Early OR Delayed) AND ("ICU-Free Days" OR "Ventilator-Free Days") | Science Direct | 30 |
| “COVID-19” AND “Respiratory Distress Syndrome” AND “Intubation" AND (Timing OR Early OR Delayed) AND ("Organ Failure-Free Days" OR "Adverse Events") | Science Direct | 188 |
| COVID19 AND Intubation | Open Grey | 0 |
| COVID19 AND Respiratory Distress Syndrome | Open Grey | 0 |
| COVID19 AND Mortality | Open Grey | 0 |
| COVID19 AND Survival | Open Grey | 0 |
| COVID19 AND Outcome | Open Grey | 0 |
| COVID19 AND Intubation | Grey Literature Report | 0 |
| COVID19 AND Respiratory Distress Syndrome | Grey Literature Report | 0 |
| COVID19 AND Mortality | Grey Literature Report | 0 |
| COVID19 AND Survival | Grey Literature Report | 0 |
| COVID19 AND Outcome | Grey Literature Report | 0 |
| **Total Articles Retrieved** | | 1280 |

**Supplementary Table S2.** Search Strategy in Cochrane Library

| ID | Search | Results |
| --- | --- | --- |
| #1 | MeSH descriptor: [COVID-19] explode all trees | 918 |
| #2 | (“COVID-19” OR “Coronavirus Disease-19” OR "2019-nCoV" OR "2019 Novel Coronavirus" OR "Coronavirus Disease 2019" OR "SARS-CoV-2" OR "SARS Coronavirus 2"):ti,ab,kw (Word variations have been searched) | 5023 |
| #3 | #1 OR #2 | 5151 |
| #4 | MeSH descriptor: [Respiratory Distress Syndrome] explode all trees | 2611 |
| #5 | MeSH descriptor: [Respiratory Insufficiency] explode all trees | 2990 |
| #6 | (“Respiratory Distress Syndrome” OR "Respiratory Insufficiency" OR "Respiratory Depression" OR "Acute Hypoxemic Respiratory Failure"):ti,ab,kw (Word variations have been searched) | 10715 |
| #7 | #4 OR #5 OR #6 | 11993 |
| #8 | MeSH descriptor: [Intubation, Intratracheal] explode all trees | 4628 |
| #9 | MeSH descriptor: [Respiration, Artificial] explode all trees | 6714 |
| #10 | MeSH descriptor: [Ventilators, Mechanical] explode all trees | 282 |
| #11 | ("Endotracheal Intubation" OR "Mechanical Ventilation" OR "Invasive Ventilation" OR "Artificial Respiration"):ti,ab,kw (Word variations have been searched) | 19315 |
| #12 | #8 OR #9 OR #10 OR #11 | 25786 |
| #13 | MeSH descriptor: [Mortality] explode all trees | 13896 |
| #14 | MeSH descriptor: [Survival] explode all trees | 133 |
| #15 | (“Mortality” OR “Mortalities” OR “Survival” OR “Survivals” OR “Survived” OR “Survives” OR “Surviving” OR “Surviveable” OR “Survivability” OR “Death” OR “Deaths” OR "ICU-Free Days" OR "Ventilator-Free Days" OR "Organ Failure-Free Days" OR "Adverse Events" OR "Adverse Outcomes" OR "Ventilation Duration" OR "ICU Length of Stay"):ti,ab,kw (Word variations have been searched) | 316319 |
| #16 | #13 OR #14 OR #15 | 316329 |
| #17 | ("Early" OR "Late" OR "delay" OR "delayed" OR "delaying" OR "delays" OR "Timely" OR "timing" OR "timings" OR "time" OR "time" OR "Prompt" OR "Rapid" OR "Quick" OR "Timely" OR "Fast" OR "Slow"):ti,ab,kw (Word variations have been searched) | 671362 |
| #18 | #3 AND #7 AND #12 AND #16 AND #17 | 131 |

**Supplementary Table S3.** Search Strategy in EMBASE Database

| **ID** | **Keywords** | **Results** |
| --- | --- | --- |
| 1 | exp coronavirus disease 2019/ | 166,139 |
| 2 | COVID-19.mp. | 179,879 |
| 3 | Coronavirus Disease-19.mp. | 2,076 |
| 4 | 2019-nCoV.mp. | 2,019 |
| 5 | 2019 Novel Coronavirus.mp. | 1,694 |
| 6 | Coronavirus Disease [2019.mp](http://2019.mp/). | 171,659 |
| 7 | exp Severe acute respiratory syndrome coronavirus 2/ | 49,421 |
| 8 | SARS-CoV-2.mp. | 70,541 |
| 9 | SARS Coronavirus [2.mp](http://2.mp/). | 3,171 |
| 10 | 1 or 2 or 3 or 4 or 5 or 6 or 7 or 8 or 9 | 212,315 |
| 11 | exp respiratory distress syndrome/ | 87,416 |
| 12 | Respiratory Distress Syndrome.mp. | 80,171 |
| 13 | Respiratory Insufficiency.mp. | 11,320 |
| 14 | Respiratory Depression.mp. | 9,759 |
| 15 | exp acute hypoxemic respiratory failure/ | 458 |
| 16 | Acute Hypoxemic Respiratory Failure.mp. | 1,316 |
| 17 | 11 or 12 or 13 or 14 or 15 or 16 | 117,889 |
| 18 | exp endotracheal intubation/ | 54,263 |
| 19 | Mechanical Ventilation.mp. or exp artificial ventilation/ | 213,979 |
| 20 | exp invasive ventilation/ | 3,433 |
| 21 | 18 or 19 or 20 | 252,149 |
| 22 | Timing.mp. | 196,579 |
| 23 | Early.mp. | 2,307,006 |
| 24 | Late.mp. | 599,916 |
| 25 | Delayed.mp. | 424,272 |
| 26 | Prompt.mp. | 102,327 |
| 27 | Rapid.mp. | 875,758 |
| 28 | Quick.mp. | 70,126 |
| 29 | 22 or 23 or 24 or 25 or 26 or 27 or 28 | 4,005,665 |
| 30 | exp mortality/ | 1,203,305 |
| 31 | [mortality.mp](http://mortality.mp/). | 1,658,284 |
| 32 | exp survival/ | 1,253,277 |
| 33 | [survival.mp](http://survival.mp/). | 1,910,628 |
| 34 | Death.mp. | 1,389,915 |
| 35 | ICU-Free Days.mp. | 309 |
| 36 | Ventilator-Free Days.mp. | 1,347 |
| 37 | Organ Failure-Free Days.mp. | 94 |
| 38 | Adverse Events.mp. | 301,205 |
| 39 | exp adverse event/ | 686,148 |
| 40 | Ventilation Duration.mp. | 1,104 |
| 41 | ICU Length of Stay.mp. | 6,319 |
| 42 | Adverse Outcomes.mp. | 40,726 |
| 43 | 30 or 31 or 32 or 33 or 34 or 35 or 36 or 37 or 38 or 39 or 40 or 41 or 42 | 4,751,849 |
| 44 | 10 and 17 and 21 and 29 and 43 | 580 |

**Supplementary Table** **S5**. Sensitivity Analysis by Excluding Each Study to Recalculate for Risk Ratio of In-Hospital Mortality between Early Intubation Compared with Late Intubation Group

| **Omitted Study** | **Risk Ratio (95% CI)** | **Heterogeneity** | | | **P value for heterogeneity** |
| --- | --- | --- | --- | --- | --- |
|  |  | **Tau^2^** | **Q** | **I^2^** |  |
| Bavishi AA (2021) | 0.96 [0.87, 1.05] | 0.02 | 36.40 | 51% | P=0.006 |
| **Vera M (2021)** | **0.98 [0.91, 1.07]** | **0.01** | **27.43** | **34%** | **P=0.07** |
| Fayed M (2021) | 0.96 [0.88, 1.06] | 0.02 | 36.22 | 50% | P=0.007 |
| Zuccon W (2021) | 0.96 [0.87, 1.05] | 0.02 | 39.01 | 54% | P=0.003 |
| Zirpe KG (2021) | 0.97 [0.88, 1.07] | 0.02 | 35.26 | 49% | P=0.009 |
| Pandya A (2020) | 0.95 [0.86, 1.05] | 0.02 | 39.89 | 55% | P=0.002 |
| Roedl K (2020) | 0.95 [0.86, 1.05] | 0.02 | 39.83 | 55% | P=0.002 |
| Siempos II (2020) | 0.95 [0.86, 1.05] | 0.02 | 40.11 | 55% | P=0.002 |
| Hyman JB (2020) | 0.95 [0.86, 1.06] | 0.02 | 36.02 | 50% | P=0.007 |
| Ferraz M (2021) | 0.95 [0.86, 1.05] | 0.02 | 40.01 | 55% | P=0.002 |
| Hernandez-Romieu AC (2020) | 0.95 [0.86, 1.05] | 0.02 | 40.09 | 55% | P=0.002 |
| Saida IB (2020) | 0.95 [0.86, 1.05] | 0.02 | 40.15 | 55% | P=0.002 |
| Dupuis C (2021) | 0.95 [0.86, 1.04] | 0.02 | 40.12 | 55% | P=0.002 |
| Mellado-Artigas R (2021) | 0.95 [0.86, 1.04] | 0.02 | 40.11 | 55% | P=0.002 |
| Karagiannidis C (2020) | 0.94 [0.84, 1.04] | 0.02 | 39.32 | 54% | P=0.003 |
| Graselli G (2020) | 0.93 [0.84, 1.04] | 0.02 | 38.95 | 54% | P=0.003 |
| Parish AJ (2021) | 0.93 [0.84, 1.03] | 0.02 | 38.06 | 53% | P=0.004 |
| COVID-ICU Group (2021) | 0.93 [0.84, 1.03] | 0.02 | 35.38 | 49% | P=0.008 |
| Matta A (2020) | 0.94 [0.85, 1.03] | 0.02 | 38.07 | 53% | P=0.004 |
| Lee YH (2020) | 0.94 [0.86, 1.04] | 0.02 | 39.05 | 54% | P=0.002 |

**Supplementary Table S6.** Sensitivity Analysis on Subgroup Analysis According to Publication Year by Excluding Each Study to Recalculate for Risk Ratio of In-Hospital Mortality between Early Intubation Compared with Late Intubation Group

| **Omitted Study** | **Risk Ratio (95% CI)** | **Heterogeneity** | | | **P value for heterogeneity** |
| --- | --- | --- | --- | --- | --- |
|  |  | **Tau^2^** | **Q** | **I^2^** |  |
| **Publication Year 2020** | | | | | |
| Pandya A (2020) | 0.98 [0.91, 1.06] | 0.00 | 8.17 | 2% | P=0.42 |
| Roedl K (2020) | 0.98 [0.91, 1.06] | 0.00 | 8.12 | 2% | P=0.42 |
| Siempos II (2020) | 0.98 [0.91, 1.06] | 0.00 | 8.35 | 4% | P=0.40 |
| Hyman JB (2020) | 1.05 [0.95, 1.16] | 0.00 | 3.42 | 0% | P=0.91 |
| Hernandez-Romieu AC (2020) | 0.98 [0.91, 1.06] | 0.00 | 8.35 | 4% | P=0.40 |
| Saida IB (2020) | 0.98 [0.91, 1.06] | 0.00 | 8.38 | 5% | P=0.40 |
| Karagiannidis C (2020) | 0.96 [0.89, 1.04] | 0.00 | 7.23 | 0% | P=0.51 |
| Graselli G (2020) | 0.96 [0.88, 1.03] | 0.00 | 6.71 | 0% | P=0.57 |
| Matta A (2020) | 0.96 [0.90, 1.04] | 0.00 | 6.11 | 0% | P=0.64 |
| Lee YH (2020) | 0.97 [0.91, 1.05] | 0.00 | 7.67 | 0% | P=0.47 |
| **Publication Year 2021** | | | | | |
| Bavishi AA (2021) | 0.86 [0.72, 1.04] | 0.04 | 28.06 | 71% | P=0.0005 |
| **Vera M (2021)** | **0.93 [0.79, 1.09]** | **0.03** | **18.78** | **57%** | **P=0.02** |
| Fayed M (2021) | 0.87 [0.72, 1.06] | 0.05 | 27.75 | 71% | P=0.0005 |
| Zuccon W (2021) | 0.85 [0.70, 1.04] | 0.05 | 30.61 | 74% | P=0.0002 |
| Zirpe KG (2021) | 0.85 [0.69, 1.05] | 0.05 | 26.15 | 69% | 0.001 |
| Ferraz M (2021) | 0.83 [0.68, 1.02] | 0.06 | 31.66 | 75% | 0.0001 |
| Dupuis C (2021) | 0.82 [0.66, 1.01] | 0.06 | 31.82 | 75% | 0.0001 |
| Mellado-Artigas R (2021) | 0.82 [0.67, 1.01] | 0.06 | 31.80 | 75% | 0.0001 |
| Parish AJ (2021) | 0.78 [0.61, 1.00] | 0.08 | 29.76 | 73% | 0.0002 |
| COVID-ICU Group (2021) | 0.78 [0.61, 0.99] | 0.08 | 25.99 | 69% | 0.001 |

**Supplementary Table** **S7**. Sensitivity Analysis on Subgroup Analysis According to Intubation Timing by Excluding Each Study to Recalculate for Risk Ratio of In-Hospital Mortality between Early Intubation Compared with Late Intubation Group

| **Omitted Study** | **Risk Ratio (95% CI)** | **Heterogeneity** | | | **P value for heterogeneity** |
| --- | --- | --- | --- | --- | --- |
|  |  | **Tau^2^** | **Q** | **I^2^** |  |
| **Intubation Timing Cut-off 24-hours** | | | | | |
| Bavishi AA (2021) | 1.04 [0.97, 1.12] | 0.00 | 9.83 | 0% | P=0.63 |
| Fayed M (2021) | 1.05 [0.97, 1.13] | 0.00 | 9.18 | 0% | P=0.69 |
| Zuccon W (2021) | 1.04 [0.96, 1.12] | 0.00 | 12.35 | 3% | P=0.42 |
| Pandya A (2020) | 1.03 [0.94, 1.12] | 0.00 | 13.32 | 10% | P=0.35 |
| Roedl K (2020) | 1.03 [0.95, 1.13] | 0.00 | 13.13 | 9% | P=0.36 |
| Siempos II (2020) | 1.02 [0.93, 1.12] | 0.00 | 13.77 | 13% | P=0.32 |
| Ferraz M (2021) | 1.02 [0.94, 1.12] | 0.00 | 13.50 | 11% | P=0.33 |
| Hernandez-Romieu AC (2020) | 1.02 [0.93, 1.12] | 0.00 | 13.63 | 12% | P=0.33 |
| Saida IB (2020) | 1.02 [0.93, 1.12] | 0.00 | 13.86 | 13% | P=0.31 |
| Mellado-Artigas R (2021) | 1.02 [0.93, 1.11] | 0.00 | 13.88 | 14% | P=0.31 |
| Karagiannidis C (2020) | 1.00 [0.90, 1.11] | 0.00 | 13.80 | 13% | P=0.31 |
| Graselli G (2020) | 1.00 [0.90, 1.11] | 0.00 | 13.69 | 12% | P=0.32 |
| COVID-ICU Group (2021) | 1.00 [0.90, 1.10] | 0.00 | 12.12 | 1% | P=0.44 |
| Lee YH (2020) | 1.02 [0.93, 1.11] | 0.00 | 13.46 | 11% | P=0.34 |
| **Intubation Timing Cut-off 48-hours** | | | | | |
| **Vera M (2021)** | **0.98 [0.91, 1.07]** | **0.01** | **27.43** | **34%** | P=**0.07** |
| Zirpe KG (2021) | 0.97 [0.88, 1.07] | 0.02 | 35.26 | 49% | P=0.0009 |
| Hyman JB (2020) | 0.95 [0.86, 1.06] | 0.02 | 36.02 | 50% | P=0.007 |
| Dupuis C (2021) | 0.95 [0.86, 1.04] | 0.02 | 40.12 | 55% | P=0.002 |
| Parish AJ (2021) | 0.93 [0.84, 1.03] | 0.02 | 38.06 | 53% | P=0.004 |
| Matta A (2020) | 0.94 [0.85, 1.03] | 0.02 | 38.07 | 53% | P=0.004 |

**Supplementary Table S8**. Sensitivity Analysis on Subgroup Analysis According to Disease Burden by Excluding Each Study to Recalculate for Risk Ratio of In-Hospital Mortality between Early Intubation Compared with Late Intubation Group

| **Omitted Study** | **Risk Ratio (95% CI)** | **Heterogeneity** | | | **P value for heterogeneity** |
| --- | --- | --- | --- | --- | --- |
|  |  | **Tau^2^** | **Q** | **I^2^** |  |
| **High Disease Burden** | | | | | |
| Fayed M (2021) | 1.00 [0.91, 1.09] | 0.01 | 17.78 | 38% | P=0.09 |
| Zuccon W (2021) | 0.99 [0.90, 1.09] | 0.01 | 20.62 | 47% | P=0.04 |
| **Zirpe KG (2021)** | **1.01 [0.93, 1.10]** | **0.01** | **16.56** | **34%** | P=**0.12** |
| Pandya A (2020) | 0.98 [0.89, 1.09] | 0.01 | 21.52 | 49% | P=0.03 |
| Hyman JB (2020) | 1.00 [0.91, 1.11] | 0.01 | 16.66 | 34% | P=0.12 |
| Hernandez-Romieu AC (2020) | 0.98 [0.89, 1.08] | 0.01 | 21.73 | 49% | P=0.03 |
| Saida IB (2020) | 0.98 [0.89, 1.08] | 0.01 | 21.82 | 50% | P=0.03 |
| Dupuis C (2021) | 0.98 [0.89, 1.08] | 0.01 | 21.80 | 50% | P=0.03 |
| Mellado-Artigas R (2021) | 0.98 [0.89, 1.08] | 0.01 | 21.78 | 50% | P=0.03 |
| Graselli G (2020) | 0.97 [0.87, 1.07] | 0.01 | 20.80 | 47% | P=0.04 |
| Parish AJ (2021) | 0.96 [0.87, 1.07] | 0.01 | 19.99 | 45% | P=0.05 |
| COVID-ICU Group (2021) | 0.96 [0.87, 1.06] | 0.01 | 17.48 | 37% | P=0.09 |
| Matta A (2020) | 0.97 [0.88, 1.06] | 0.01 | 19.86 | 45% | P=0.05 |
| **Low Disease Burden** | | | | | |
| Bavishi AA (2021) | 0.86 [0.63, 1.16] | 0.08 | 14.28 | 65% | P=0.01 |
| **Vera M (2021)** | **0.99 [0.83, 1.18]** | **0.01** | **5.57** | **10%** | P=**0.35** |
| Roedl K (2020) | 0.78 [0.52, 1.18] | 0.16 | 17.70 | 72% | P=0.003 |
| Siempos II (2020) | 0.80 [0.56, 1.13] | 0.12 | 17.81 | 72% | P=0.003 |
| Ferraz M (2021) | 0.78 [0.53, 1.16] | 0.14 | 17.79 | 72% | P=0.003 |
| Karagiannidis C (2020) | 0.74 [0.50, 1.10] | 0.13 | 12.57 | 60% | P=0.03 |
| Lee YH (2020) | 0.75 [0.53, 1.08] | 0.12 | 16.90 | 70% | P=0.005 |

**Supplementary Table S9.** Sensitivity Analysis on Studies with Low to Moderate Risk of Bias by Excluding Each Study to Recalculate for Risk Ratio of In-Hospital Mortality between Early Intubation Compared with Late Intubation Group

| **Omitted Study** | **Risk Ratio (95% CI)** | **Heterogeneity** | | | **P value for heterogeneity** |
| --- | --- | --- | --- | --- | --- |
|  |  | **Tau^2^** | **Q** | **I^2^** |  |
| Bavishi AA (2021) | 0.79 [0.63, 0.99] | 0.05 | 13.13 | 47% | P=0.07 |
| **Vera M (2021)** | **0.83 [0.69, 1.00]** | **0.01** | **8.36** | **16%** | **P=0.30** |
| Fayed M (2021) | 0.79 [0.62, 1.02] | 0.06 | 14.26 | 51% | P=0.05 |
| Zirpe KG (2021) | 0.75 [0.55, 1.03] | 0.10 | 15.78 | 56% | P=0.03 |
| Pandya A (2020) | 0.75 [0.57, 0.98] | 0.07 | 15.50 | 55% | P=0.03 |
| Siempos II (2020) | 0.76 [0.59, 0.98] | 0.07 | 15.71 | 55% | P=0.03 |
| Hernandez-Romieu AC (2020) | 0.74 [0.56, 0.97] | 0.07 | 15.09 | 54% | P=0.03 |
| Dupuis C (2021) | 0.73 [0.56, 0.94] | 0.06 | 13.82 | 49% | P=0.05 |
| Lee YH (2020) | 0.73 [0.57, 0.93] | 0.05 | 13.37 | 48% | P=0.06 |

**Supplementary Table** **S10**. Sensitivity Analysis on Studies with Comparable SOFA Score between Groups by Excluding Each Study to Recalculate for Risk Ratio of In-Hospital Mortality between Early Intubation Compared with Late Intubation Group

| **Omitted Study** | **Risk Ratio (95% CI)** | **Heterogeneity** | | | **P value for heterogeneity** |
| --- | --- | --- | --- | --- | --- |
|  |  | **Tau^2^** | **Q** | **I^2^** |  |
| Bavishi AA (2021) | 0.79 [0.60, 1.04] | 0.02 | 3.80 | 21% | P=0.28 |
| Fayed M (2021) | 0.82 [0.54, 1.25] | 0.07 | 4.92 | 39% | P=0.18 |
| Zirpe KG (2021) | 0.71 [0.38, 1.31] | 0.20 | 6.38 | 53% | P=0.09 |
| Siempos II (2020) | 0.73 [0.47, 1.11] | 0.09 | 6.32 | 53% | P=0.10 |
| **Lee YH (2020)** | **0.69 [0.50, 0.94]** | **0.03** | **3.77** | **20%** | **P=0.29** |

**Supplementary Table S11**. Sensitivity Analysis on Studies with Comparable P_a_O_2_/F_I_O_2_ Ratio between Groups by Excluding Each Study to Recalculate for Risk Ratio of In-Hospital Mortality between Early Intubation Compared with Late Intubation Group

| **Omitted Study** | **Risk Ratio (95% CI)** | **Heterogeneity** | | | **P value for heterogeneity** |
| --- | --- | --- | --- | --- | --- |
|  |  | **Tau^2^** | **Q** | **I^2^** |  |
| Bavishi AA (2021) | 0.90 [0.71, 1.13] | 0.06 | 17.80 | 61% | P=0.01 |
| **Vera M (2021)** | **0.95 [0.79, 1.15]** | **0.02** | **10.79** | **35%** | **P=0.15** |
| Zirpe KG (2021) | 0.88 [0.66, 1.18] | 0.10 | 19.10 | 63% | P=0.008 |
| Pandya A (2020) | 0.86 [0.66, 1.13] | 0.09 | 21.00 | 67% | P=0.004 |
| Hernandez-Romieu AC (2020) | 0.86 [0.65, 1.12] | 0.09 | 20.93 | 67% | P=0.004 |
| Dupuis C (2021) | 0.85 [0.65, 1.11] | 0.09 | 20.38 | 66% | P=0.005 |
| Mellado-Artigas R (2021) | 0.85 [0.65, 1.10] | 0.09 | 20.41 | 66% | P=0.005 |
| Matta A (2020) | 0.82 [0.64, 1.04] | 0.06 | 15.43 | 55% | P=0.03 |
| Lee YH (2020) | 0.84 [0.65, 1.08] | 0.08 | 19.61 | 64% | P=0.006 |

**Supplementary Table S12**. Sensitivity Analysis by Excluding Each Study to Recalculate for Mean Difference of ICU Length of Stay between Early Intubation Compared with Late Intubation Group

| **Omitted Study** | **Mean Difference (95% CI)** | **Heterogeneity** | | | **P value for heterogeneity** |
| --- | --- | --- | --- | --- | --- |
|  |  | **Tau^2^** | **Q** | **I^2^** |  |
| Lee YH (2020) | -2.03 [-4.25, 0.18] | 8.10 | 28.86 | 72% | P=0.0003 |
| **Vera M (2021)** | **-1.48 [-3.58, 0.62]** | **6.09** | **22.56** | **65%** | **P=0.004** |
| Pandya A (2020) | -1.68 [-3.98, 0.62] | 7.61 | 25.05 | 68% | P=0.002 |
| Bavishi AA (2021) | -2.14 [-4.66, 0.39] | 10.14 | 32.01 | 75% | P<0.0001 |
| Karagiannidis C (2020) | -2.13 [-4.71, 0.46] | 10.50 | 31.56 | 75% | P=0.0001 |
| Dupuis C (2021) | -2.47 [-5.14, 0.21] | 11.40 | 32.34 | 75% | P<0.0001 |
| Hernandez-Romieu AC (2020) | -2.54 [-5.20, 0.12] | 11.20 | 31.66 | 75% | P=0.0001 |
| Zirpe KG (2021) | -2.57 [-5.17, 0.02] | 10.58 | 31.36 | 74% | P=0.0001 |
| Fayed M (2021) | -2.59 [-5.05, -0.13] | 9.58 | 31.21 | 74% | P=0.0001 |
| Matta A (2020) | -2.85 [-5.11, -0.58] | 7.20 | 23.62 | 66% | P=0.003 |

**Supplementary Table S13**. Sensitivity Analysis on Subgroup Analysis According to Publication Year by Excluding Each Study to Recalculate for Mean Difference of ICU Length of Stay between Early Intubation Compared with Late Intubation Group

| **Omitted Study** | **Mean Difference (95% CI)** | **Heterogeneity** | | | **P value for heterogeneity** |
| --- | --- | --- | --- | --- | --- |
|  |  | **Tau^2^** | **Q** | **I^2^** |  |
| **Publication Year 2020** | | | | | |
| Lee YH (2020) | -1.79 [-5.42, 1.84] | 11.13 | 16.08 | 81% | P=0.001 |
| Pandya A (2020) | -0.95 [-4.82, 2.92] | 9.48 | 10.84 | 72% | P=0.01 |
| Karagiannidis C (2020) | -2.22 [-7.33, 2.88] | 18.64 | 18.50 | 84% | P=0.0003 |
| Hernandez-Romieu AC (2020) | -3.31 [-8.82, 2.20] | 21.92 | 18.97 | 84% | P=0.0003 |
| **Matta A (2020)** | **-3.77 [-7.63, 0.08]** | **9.21** | **10.06** | **70%** | **P=0.02** |
| **Publication Year 2021** | | | | | |
| **Vera M (2021)** | **-0.81 [-2.61, 0.99]** | **0.00** | **2.26** | **0%** | **P=0.52** |
| Bavishi AA (2021) | -2.05 [-5.89, 1.78] | 11.23 | 12.11 | 75% | P=0.007 |
| Dupuis C (2021) | -2.72 [-7.01, 1.57] | 14.24 | 12.12 | 75% | P=0.007 |
| Zirpe KG (2021) | -2.97 [-6.93, 0.98] | 11.58 | 10.85 | 72% | P=0.01 |
| Fayed M (2021) | -3.02 [-6.54, 0.51] | 9.24 | 11.00 | 73% | P=0.01 |

**Supplementary Table S14**. Sensitivity Analysis on Subgroup Analysis According to Disease Burden by Excluding Each Study to Recalculate for Mean Difference of ICU Length of Stay between Early Intubation Compared with Late Intubation Group

| **Omitted Study** | **Mean Difference (95% CI)** | **Heterogeneity** | | | **P value for heterogeneity** |
| --- | --- | --- | --- | --- | --- |
|  |  | **Tau^2^** | **Q** | **I^2^** |  |
| **High Disease Burden** | | | | | |
| **Pandya A (2020)** | **0.31 [-1.11, 1.73]** | **0.00** | **3.40** | **0%** | **P=0.09** |
| Dupuis C (2021) | -0.60 [-3.56, 2.35] | 8.25 | 15.37 | 74% | P=0.004 |
| Hernandez-Romieu AC (2020) | -0.73 [-3.71, 2.26] | 8.39 | 15.41 | 74% | P=0.004 |
| Zirpe KG (2021) | -0.82 [-3.70, 2.06] | 7.77 | 15.24 | 74% | P=0.004 |
| Fayed M (2021) | -0.95 [-3.57, 1.68] | 6.52 | 14.83 | 73% | P=0.005 |
| Matta A (2020) | -1.40 [-3.76, 0.97] | 4.27 | 10.02 | 60% | P=0.04 |
| **Low Disease Burden** | | | | | |
| Lee YH (2020) | -5.05 [-8.48, -1.61] | 4.81 | 4.18 | 52% | P=0.12 |
| **Vera M (2021)** | **-3.97 [-8.00, 0.06]** | **4.77** | **3.23** | **38%** | **P=0.20** |
| Karagiannidis C (2020) | -7.36 [-13.82, -0.91] | 17.74 | 5.50 | 64% | P=0.06 |
| Bavishi AA (2021) | -7.21 [-13.57, -0.85] | 18.08 | 6.35 | 69% | P=0.04 |

**Supplementary Table S15**. Sensitivity Analysis on Subgroup Analysis According to Intubation Timing by Excluding Each Study to Recalculate for Mean Difference of ICU Length of Stay between Early Intubation Compared with Late Intubation Group

| **Omitted Study** | **Mean Difference (95% CI)** | **Heterogeneity** | | | **P value for heterogeneity** |
| --- | --- | --- | --- | --- | --- |
|  |  | **Tau^2^** | **Q** | **I^2^** |  |
| **Intubation Timing Cut-off 24-hours** | | | | | |
| Lee YH (2020) | -2.59 [-5.09, -0.09] | 4.48 | 9.19 | 56% | P=0.06 |
| **Pandya A (2020)** | **-1.88 [-4.62, 0.87]** | **4.12** | **7.40** | **46%** | **P=0.12** |
| Karagiannidis C (2020) | -2.79 [-6.44, 0.87] | 10.20 | 12.46 | 68% | P=0.01 |
| Bavishi AA (2021) | -2.78 [-6.22, 0.66] | 9.08 | 12.56 | 68% | P=0.01 |
| Hernandez-Romieu AC (2020) | -3.59 [-6.81, -0.38] | 6.70 | 8.98 | 55% | P=0.06 |
| Fayed M (2021) | -3.57 [-6.53, -0.61] | 6.10 | 10.08 | 60% | P=0.04 |
| **Intubation Timing Cut-off 48-hours** | | | | | |
| **Vera M (2021)** | **0.50 [-1.62, 2.62]** | **1.12** | **2.94** | **32%** | **P=0.23** |
| Dupuis C (2021) | -1.89 [-7.86, 4.07] | 24.29 | 16.92 | 88% | P=0.0002 |
| Zirpe KG (2021) | -2.21 [-7.93, 3.51] | 22.23 | 16.75 | 88% | P=0.0002 |
| Matta A (2020) | -3.01 [-7.68, 1.66] | 13.68 | 10.69 | 81% | P=0.005 |

**Supplementary Table S16**. Sensitivity Analysis on Studies with Low to Moderate Risk of Bias by Excluding Each Study to Recalculate for ICU Length of Stay between Early Intubation Compared with Late Intubation Group

| **Omitted Study** | **Risk Ratio (95% CI)** | **Heterogeneity** | | | **P value for heterogeneity** |
| --- | --- | --- | --- | --- | --- |
|  |  | **Tau^2^** | **Q** | **I^2^** |  |
| Lee YH (2020) | -2.56 [-5.03, -0.09] | 7.48 | 19.66 | 69% | P=0.003 |
| **Vera M (2021)** | **-1.87 [-4.20, 0.45]** | **5.19** | **14.50** | **59%** | **P=0.02** |
| Pandya A (2020) | -2.17 [-4.84, 0.50] | 7.52 | 17.22 | 65% | P=0.009 |
| Bavishi AA (2021) | -2.78 [-5.75, 0.20] | 10.51 | 22.98 | 74% | P=0.0008 |
| Dupuis C (2021) | -3.24 [-6.38, -0.09] | 11.68 | 22.30 | 73% | P=0.001 |
| Hernandez-Romieu AC (2020) | -3.33 [-6.40, -0.25] | 10.98 | 21.11 | 72% | P=0.002 |
| Zirpe KG (2021) | -3.36 [-6.34, -0.38] | 10.21 | 20.92 | 71% | P=0.002 |
| Fayed M (2021) | -3.35 [-6.15, -0.55] | 9.12 | 21.24 | 72% | P=0.002 |

**Supplementary Table S17**. Sensitivity Analysis on Studies with Comparable SOFA between Groups by Excluding Each Study to Recalculate for Mean Difference of ICU Length of Stay between Early Intubation Compared with Late Intubation Group

| **Omitted Study** | **Mean Difference (95% CI)** | **Heterogeneity** | | | **P value for heterogeneity** |
| --- | --- | --- | --- | --- | --- |
|  |  | **Tau^2^** | **Q** | **I^2^** |  |
| **Lee YH (2020)** | **-0.70 [-3.17, 1.77]** | **0.53** | **2.23** | **10%** | **P=0.33** |
| Bavishi AA (2021) | -0.59 [-6.01, 4.82] | 11.46 | 4.53 | 56% | P=0.10 |
| Zirpe KG (2021) | -2.80 [-9.68, 4.08] | 20.48 | 5.66 | 65% | P=0.06 |
| Fayed M (2021) | -2.66 [-8.13, 2.81] | 12.52 | 5.38 | 63% | P=0.07 |

**Supplementary Table** **S18**. Sensitivity Analysis on Studies with Comparable P_a_O_2_/F_I_O_2_ Score between Groups by Excluding Each Study to Recalculate for Mean Difference of ICU Length of Stay between Early Intubation Compared with Late Intubation Group

| **Omitted Study** | **Risk Ratio (95% CI)** | **Heterogeneity** | | | **P value for heterogeneity** |
| --- | --- | --- | --- | --- | --- |
|  |  | **Tau^2^** | **Q** | **I^2^** |  |
| Lee YH (2020) | -2.23 [-4.91, 0.44] | 9.90 | 26.56 | 77% | P=0.0002 |
| **Vera M (2021)** | **-1.53 [-4.07, 1.01]** | **7.42** | **20.01** | **70%** | **P=0.003** |
| Pandya A (2020) | -1.85 [-4.67, 0.97] | 9.43 | 22.41 | 73% | P=0.001 |
| Bavishi AA (2021) | -2.46 [-5.59, 0.68] | 12.69 | 29.68 | 80% | P<0.0001 |
| Dupuis C (2021) | -2.92 [-6.29, 0.45] | 14.70 | 30.08 | 80% | P<0.0001 |
| Hernandez-Romieu AC (2020) | -3.02 [-6.37, 0.34] | 14.48 | 29.41 | 80% | P<0.0001 |
| Zirpe KG (2021) | -3.04 [-6.30, 0.21] | 13.54 | 29.11 | 79% | P<0.0001 |
| Matta A (2020) | -3.35 [-6.15, -0.55] | 9.12 | 21.24 | 72% | P=0.002 |

**Supplementary Table S19.** Sensitivity Analysis by Excluding Each Study to Recalculate for Mean Difference of Ventilation Duration between Early Intubation Compared with Late Intubation Group

| **Omitted Study** | **Mean Difference (95% CI)** | **Heterogeneity** | | | **P value for heterogeneity** |
| --- | --- | --- | --- | --- | --- |
|  |  | **Tau^2^** | **Q** | **I^2^** |  |
| Lee YH (2020) | -0.20 [-1.96, 1.56] | 3.92 | 19.66 | 59% | P=0.01 |
| **Pandya A (2020)** | **0.16 [-1.61, 1.93]** | **3.33** | **17.03** | **53%** | **P=0.03** |
| Vera M (2021) | 0.04 [-1.82, 1.89] | 4.00 | 19.10 | 58% | P=0.01 |
| Karagiannidis C (2020) | -0.04 [-2.05, 1.98] | 4.69 | 18.57 | 57% | P=0.02 |
| Bavishi AA (2021) | -0.18 [-2.13, 1.78] | 4.80 | 21.42 | 63% | P=0.006 |
| Zirpe KG (2021) | -0.58 [-2.71, 1.55] | 5.61 | 20.69 | 61% | P=0.008 |
| Hernandez-Romieu AC (2020) | -0.58 [-2.70, 1.53] | 5.53 | 20.70 | 61% | P=0.008 |
| Siempos II (2020) | -0.46 [-2.39, 1.47] | 4.77 | 21.72 | 63% | P=0.005 |
| Matta A (2020) | -0.72 [-2.65, 1.22] | 4.31 | 19.05 | 58% | P=0.01 |
| Fayed M (2021) | -0.73 [-2.50, 1.05] | 3.54 | 17.98 | 56% | P=0.02 |

**Supplementary Table S20.** Sensitivity Analysis on Subgroup Analysis According to Publication Year by Excluding Each Study to Recalculate for Mean Difference of ICU Length of Stay Ventilation Duration between Early Intubation Compared with Late Intubation Group

| **Omitted Study** | **Mean Difference (95% CI)** | **Heterogeneity** | | | **P value for heterogeneity** |
| --- | --- | --- | --- | --- | --- |
|  |  | **Tau^2^** | **Q** | **I^2^** |  |
| **Publication Year 2020** | | | | | |
| Lee YH (2020) | -0.36 [-2.72, 2.01] | 4.37 | 11.27 | 65% | P=0.02 |
| **Pandya A (2020)** | **0.25 [-2.18, 2.68]** | **3.62** | **8.98** | **55%** | **P=0.06** |
| Karagiannidis C (2020) | -0.18 [-3.30, 2.94] | 6.71 | 10.56 | 62% | P=0.03 |
| Hernandez-Romieu AC (2020) | -1.15 [-4.34, 2.04] | 7.22 | 11.24 | 64% | P=0.02 |
| Siempos II (2020) | -0.87 [-3.62, 1.88] | 5.89 | 13.14 | 70% | P=0.01 |
| Matta A (2020) | -1.36 [-4.04, 1.33] | 4.59 | 9.64 | 59% | P=0.05 |
| **Publication Year 2021** | | | | | |
| **Vera M (2021)** | **1.16 [-1.96, 4.27]** | **3.78** | **3.89** | **49%** | **P=0.14** |
| Bavishi AA (2021) | 0.58 [-3.59, 4.75] | 9.53 | 6.93 | 71% | P=0.03 |
| Zirpe KG (2021) | -0.45 [-5.65, 4.75] | 15.12 | 7.08 | 72% | P=0.03 |
| Fayed M (2021) | -1.14 [-4.32, 2.04] | 4.35 | 4.39 | 54% | P=0.11 |

**Supplementary Table S21.** Sensitivity Analysis on Subgroup Analysis According to Intubation Timing by Excluding Each Study to Recalculate for Mean Difference of Ventilation Duration between Early Intubation Compared with Late Intubation Group

| **Omitted Study** | **Mean Difference (95% CI)** | **Heterogeneity** | | | **P value for heterogeneity** |
| --- | --- | --- | --- | --- | --- |
|  |  | **Tau^2^** | **Q** | **I^2^** |  |
| **Intubation Timing Cut-off 24-hours** | | | | | |
| Lee YH (2020) | -0.47 [-2.82, 1.89] | 4.77 | 12.67 | 61% | P=0.03 |
| Pandya A (2020) | 0.07 [-2.44, 2.58] | 4.46 | 10.90 | 54% | P=0.05 |
| Karagiannidis C (2020) | -0.31 [-3.47, 2.85] | 8.29 | 12.82 | 61% | P=0.03 |
| Bavishi AA (2021) | -0.45 [-3.27, 2.38] | 6.78 | 14.58 | 66% | P=0.01 |
| Hernandez-Romieu AC (2020) | -1.15 [-4.10, 1.81] | 6.78 | 11.53 | 57% | P=0.04 |
| Siempos II (2020) | -0.93 [-3.61, 1.76] | 6.23 | 14.34 | 65% | P=0.01 |
| **Fayed M (2021)** | **-1.41 [-3.65, 0.84]** | **3.32** | **9.79** | **49%** | **P=0.08** |
| **Intubation Timing Cut-off 48-hours** | | | | | |
| **Vera M (2021)** | **1.48 [-0.32, 3.28]** | **0.00** | **0.53** | **0%** | **P=0.47** |
| Zirpe KG (2021) | -0.58 [-6.84, 5.69] | 16.64 | 5.27 | 81% | P=0.02 |
| Matta A (2020) | -1.10 [-5.93, 3.74] | 9.20 | 3.79 | 74% | P=0.05 |

**Supplementary Table S22**. Sensitivity Analysis on Subgroup Analysis According to Disease Burden by Excluding Each Study to Recalculate for Mean Difference of Ventilation Duration between Early Intubation Compared with Late Intubation Group

| **Omitted Study** | **Mean Difference (95% CI)** | **Heterogeneity** | | | **P value for heterogeneity** |
| --- | --- | --- | --- | --- | --- |
|  |  | **Tau^2^** | **Q** | **I^2^** |  |
| **High Disease Burden** | | | | | |
| **Pandya A (2020)** | **1.58 [0.21, 2.95]** | **0.00** | **2.45** | **0%** | **P=0.48** |
| Zirpe KG (2021) | 0.86 [-2.36, 4.08] | 7.48 | 10.63 | 72% | P=0.01 |
| Hernandez-Romieu AC (2020) | 0.85 [-2.34, 4.04] | 7.32 | 10.62 | 72% | P=0.01 |
| Matta A (2020) | 0.49 [-2.28, 3.26] | 5.18 | 9.55 | 69% | P=0.02 |
| Fayed M (2021) | 0.33 [-1.94, 2.60] | 3.29 | 8.04 | 63% | P=0.05 |
| **Low Disease Burden** | | | | | |
| Lee YH (2020) | -2.02 [-3.76, -0.27] | 0.00 | 2.03 | 0% | P=0.57 |
| Vera M (2021) | -1.77 [-3.77, 0.22] | 0.19 | 3.09 | 3% | P=0.38 |
| Karagiannidis C (2020) | -2.30 [-5.71, 1.12] | 2.70 | 3.84 | 22% | P=0.28 |
| Bavishi AA (2021) | -2.19 [-4.82, 0.43] | 1.83 | 3.86 | 22% | P=0.28 |
| Siempos II (2020) | -2.44 [-4.25, -0.62] | 0.00 | 2.38 | 0% | P=0.50 |

**Supplementary Table S23.** Sensitivity Analysis on Studies with Low to Moderate Risk of Bias by Excluding Each Study to Recalculate for Ventilation Duration between Early Intubation Compared with Late Intubation Group

| **Omitted Study** | **Risk Ratio (95% CI)** | **Heterogeneity** | | | **P value for heterogeneity** |
| --- | --- | --- | --- | --- | --- |
|  |  | **Tau^2^** | **Q** | **I^2^** |  |
| Lee YH (2020) | -0.28 [-2.45, 1.89] | 4.50 | 14.18 | 58% | P=0.03 |
| **Pandya A (2020)** | **0.27 [-1.85, 2.39]** | **3.21** | **10.95** | **45%** | **P=0.09** |
| Vera M (2021) | 0.05 [-2.26, 2.36] | 4.53 | 13.30 | 55% | P=0.04 |
| Bavishi AA (2021) | -0.29 [-2.80, 2.23] | 6.05 | 15.85 | 62% | P=0.01 |
| Zirpe KG (2021) | -0.90 [-3.78, 1.98] | 8.16 | 15.50 | 61% | P=0.02 |
| Hernandez-Romieu AC (2020) | -0.90 [-3.77, 1.96] | 8.03 | 15.50 | 61% | P=0.02 |
| Siempos II (2020) | -0.70 [-3.17, 1.77] | 6.05 | 16.35 | 63% | P=0.01 |
| Fayed M (2021) | -1.07 [-3.29, 1.15] | 4.08 | 12.76 | 53% | P=0.05 |

**Supplementary Table S24.** Sensitivity Analysis on Studies with Comparable SOFA between Groups by Excluding Each Study to Recalculate for Mean Difference of Ventilation Duration between Early Intubation Compared with Late Intubation Group

| **Omitted Study** | **Mean Difference (95% CI)** | **Heterogeneity** | | | **P value for heterogeneity** |
| --- | --- | --- | --- | --- | --- |
|  |  | **Tau^2^** | **Q** | **I^2^** |  |
| **Lee YH (2020)** | **1.17 [-1.15, 3.48]** | **1.43** | **3.91** | **23%** | **P=0.27** |
| Bavishi AA (2021) | 1.71 [-1.66, 5.08] | 4.51 | 4.87 | 38% | P=0.18 |
| Zirpe KG (2021) | 0.52 [-4.44, 5.48] | 12.85 | 6.65 | 55% | P=0.08 |
| Siempos II (2020) | 0.65 [-3.06, 4.36] | 7.04 | 6.61 | 55% | P=0.09 |
| Fayed M (2021) | 0.05 [-2.70, 2.79] | 2.13 | 3.96 | 24% | P=0.27 |

**Supplementary Table S25.** Sensitivity Analysis on Studies with Comparable P_a_O_2_/F_I_O_2_ Score between Groups by Excluding Each Study to Recalculate for Mean Difference of Ventilation Duratoin between Early Intubation Compared with Late Intubation Group

| **Omitted Study** | **Mean Difference (95% CI)** | **Heterogeneity** | | | **P value for heterogeneity** |
| --- | --- | --- | --- | --- | --- |
|  |  | **Tau^2^** | **Q** | **I^2^** |  |
| Lee YH (2020) | -0.50 [-2.59, 1.59] | 3.88 | 12.59 | 60% | P=0.03 |
| **Pandya A (2020)** | **0.11 [-1.89, 2.11]** | **2.58** | **9.33** | **46%** | **P=0.10** |
| Vera M (2021) | -0.19 [-2.39, 2.02] | 3.83 | 11.69 | 57% | P=0.04 |
| Bavishi AA (2021) | -0.59 [-3.03, 1.84] | 5.24 | 14.25 | 65% | P=0.01 |
| Zirpe KG (2021) | -1.34 [-4.16, 1.48] | 7.02 | 14.02 | 64% | P=0.02 |
| Hernandez-Romieu AC (2020) | -1.34 [-4.14, 1.46] | 6.90 | 14.02 | 64% | P=0.02 |
| Matta A (2020) | -1.40 [-3.85, 1.04] | 4.80 | 12.38 | 60% | P=0.03 |

**Supplementary Table S26.** Sensitivity Analysis by Excluding Each Study to Recalculate for Mean Difference of Ventilator Free Days between Early Intubation Compared with Late Intubation Group

| **Omitted Study** | **Mean Difference (95% CI)** | **Heterogeneity** | | | **P value for heterogeneity** |
| --- | --- | --- | --- | --- | --- |
|  |  | **Tau^2^** | **Q** | **I^2^** |  |
| Lee YH (2020) | -0.04 [-3.76, 3.67] | 8.22 | 9.12 | 78% | P=0.01 |
| **Dupuis C (2021)** | **0.21 [-5.09, 5.51]** | **11.84** | **5.04** | **60%** | **P=0.08** |
| Siempos II (2020) | -1.89 [-7.21, 3.43] | 14.26 | 9.07 | 78% | P=0.01 |
| Vera M (2021) | -2.12 [-6.75, 2.51] | 10.00 | 6.48 | 69% | P=0.04 |

**Supplementary Table S27.** Sensitivity Analysis by Excluding Each Study to Recalculate for Risk Ratio of Continuous Renal Replacement Therapy between Early Intubation Compared with Late Intubation Group

| **Omitted Study** | **Risk Ratio (95% CI)** | **Heterogeneity** | | | **P value for heterogeneity** |
| --- | --- | --- | --- | --- | --- |
|  |  | **Tau^2^** | **Q** | **I^2^** |  |
| **Siempos II (2020)** | **0.95 [0.46, 1.96]** | **0.20** | **4.05** | **51%** | **P=0.13** |
| Bavishi AA (2021) | 0.76 [0.27, 2.12] | 0.66 | 10.06 | 80% | P=0.007 |
| Vera M (2021) | 0.52 [0.15, 1.82] | 0.97 | 10.47 | 81% | P=0.005 |
| Matta A (2020) | 0.47 [0.17, 1.28] | 0.55 | 6.79 | 71% | P=0.03 |

**Supplementary Table S28.** Risk Of Bias In Non-randomised Studies - of Interventions (ROBINS-I)

| Study | | Pre-Intervention | | At Intervention | Post-Intervention | | | | The overall risk of bias |
| --- | --- | --- | --- | --- | --- | --- | --- | --- | --- |
| First Author | Year | Bias due to confounding | Bias in the selection of participants into the study | Bias in the classification of intervention | Bias due to deviations from intended interventions | Bias due to missing data | Bias in the measurement of outcomes | Bias in the selection of the reported result | Low / moderate / serious / critical |
| Bavishi, et al. | 2021 | Moderate | Moderate | Moderate | Low | Low | Low | Low | Moderate |
| COVID-ICU Group | 2021 | Serious | Low | Low | Low | Low | Low | Low | Serious |
| Dupuis, et al. | 2021 | Moderate | Low | Low | Low | Low | Low | Low | Moderate |
| Fayed, et al. | 2021 | Low | Moderate | Moderate | Low | Low | Low | Low | Moderate |
| Ferraz, et al. | 2021 | Critical | Moderate | Moderate | Low | Low | Low | Low | Critical |
| Grasselli, et al. | 2020 | Serious | Moderate | Moderate | Low | Low | Low | Low | Serious |
| Hernandez-Romieu, et al. | 2020 | Moderate | Moderate | Moderate | Low | Low | Low | Low | Moderate |
| Hyman, et al. | 2020 | Serious | Moderate | Moderate | Low | Low | Low | Low | Serious |
| Karagiannidis, et al. | 2020 | Serious | Moderate | Moderate | Low | Low | Low | Low | Serious |
| Lee, et al. | 2020 | Low | Moderate | Moderate | Low | Low | Low | Low | Moderate |
| Matta, et al. | 2020 | Serious | Moderate | Moderate | Low | Low | Low | Low | Serious |
| Mellado-Artigas, et al. | 2021 | Serious | Low | Low | Low | Low | Low | Low | Serious |
| Pandya, et al. | 2021 | Moderate | Moderate | Moderate | Low | Low | Low | Low | Moderate |
| Parish, et al. | 2021 | Serious | Moderate | Moderate | Low | Low | Low | Low | Serious |
| Roedl, et al. | 2021 | Serious | Moderate | Moderate | Low | Low | Low | Low | Serious |
| Saida, et al. | 2021 | Serious | Moderate | Moderate | Low | Low | Low | Low | Serious |
| Siempos, et al. | 2020 | Moderate | Moderate | Moderate | Low | Low | Low | Low | Moderate |
| Vera, et al. | 2021 | Moderate | Low | Low | Low | Low | Low | Low | Moderate |
| Zirpe, et al. | 2021 | Moderate | Moderate | Moderate | Low | Low | Low | Low | Moderate |
| Zuccon, et al. | 2021 | Serious | Moderate | Moderate | Low | Low | Low | Low | Serious |

**Bias due to confounding**

Low: No confounding expected

Moderate: Confounding expected, all known important confounding domains appropriately measured and controlled for and reliability and validity of measurement of important domains were sufficient, such that we do not expect serious residual confounding.

Serious: At least one known important domain was not appropriately measured, or not controlled for

Critical: Confounding inherently not controllable

**Bias in the selection of participants into the study**

Low: All participants who would have been eligible for the target trial were included in the study and for each participant, start of follow up and start of intervention coincided.

Moderate: Selection into the study may have been related to intervention and outcome and the authors used appropriate methods to adjust for the selection bias;

**Bias in the classification of intervention**

Low: Intervention status is well defined intervention definition is based solely on information collected at the time of intervention.

Moderate: Intervention status is well defined and some aspects of the assignments of intervention status were determined retrospectively.

**Bias due to deviations from intended interventions**

Low: Effect of assignment to intervention showed that any deviations from intended intervention reflected usual practice or any deviations from usual practice were unlikely to impact on the outcome. Effect of starting and adhering to intervention showed that the important co-interventions were balanced across intervention groups, and there were no deviations from the intended interventions (in terms of implementation or adherence) that were likely to impact on the outcome

**Bias due to missing data**

Low: Data were reasonably complete

**Bias in the measurement of outcomes**

Low: The methods of outcome assessment were comparable across intervention groups and the outcome measure was unlikely to be influenced by knowledge of the intervention received by study participants (i.e. is objective) or the outcome assessors were unaware of the intervention received by study participants and any error in measuring the outcome is unrelated to intervention status.

**Bias in the selection of the reported result**

Low: There is clear evidence that all reported results correspond to all intended outcomes, analyses and sub-cohorts.
