# Supplementary material for: Outcome in early vs late intubation among COVID-19 patients with acute respiratory distress syndrome: an updated systematic review and meta-analysis
Source: Sci Rep. 2022 Dec 14;12:21588. doi: 10.1038/s41598-022-26234-7 (PMC9748395; doi:10.1038/s41598-022-26234-7)
Supplement: Supplementary file 4 — Supplementary Information 4. [file 41598_2022_26234_MOESM4_ESM.docx]

**Supplementary Table S29**

**Manuscript Title:** Outcome in Early vs Late Intubation among COVID-19 Patients with Acute Respiratory Distress Syndrome: An Updated Systematic Review and Meta-Analysis

**Author List:** Denio A. Ridjab^1^, Ignatius Ivan^2^, Fanny Budiman^2^, Dafsah A. Juzar^3^

^1^Department of Medical Education Unit, School of Medicine and Health Sciences, Atma Jaya Catholic University of Indonesia, Jakarta, Indonesia

^2^School of Medicine and Health Sciences, Atma Jaya Catholic University of Indonesia, Jakarta, Indonesia

^3^Department of Cardiology and Vascular Medicine, Faculty of Medicine, Universitas Indonesia/Harapan Kita National Cardiovascular Centre

**Supplementary Table S29.** Certainty in Overall Effect Estimates Using the Grading of Recommendations Assessment, Development and Evaluation (GRADE) Methods

**Author(s):** Denio A. Ridjab, Ignatius Ivan, Fanny Budiman, Dafsah Arifa Juzar

**Question:** Early Intubation compared to Late Intubation for COVID-19 Patients with Acute Respiratory Distress Syndrome

**Setting:** Intensive Care Unit

**Bibliography:**

| **Certainty assessment** | | | | | | | **№ of patients** | | **Effect** | | **Certainty** | **Importance** |
| --- | --- | --- | --- | --- | --- | --- | --- | --- | --- | --- | --- | --- |
| **№ of studies** | **Study design** | **Risk of bias** | **Inconsistency** | **Indirectness** | **Imprecision** | **Other considerations** | **Early Intubation** | **Late Intubation** | **Relative (95% CI)** | **Absolute (95% CI)** |  |  |
| **In-Hospital Mortality** | | | | | | | | | | | | |
| 9^a^ | observational studies | not serious | serious^b^ | not serious^c^ | not serious^d^ | none | 204/560 (36.4%) | 197/397 (49.6%) | **RR 0.76** (0.60 to 0.97) | **119 fewer per 1,000** (from 198 fewer to 15 fewer) | ⨁⨁⨁◯ Moderate | CRITICAL |
| **ICU Length of Stay** | | | | | | | | | | | | |
| 8^a^ | observational studies | not serious | serious^e^ | not serious^c^ | serious^f^ | none | 541 | 383 | - | MD **2.81 lower** (5.42 lower to 0.2 lower) | ⨁◯◯◯ Very low | IMPORTANT |
| **Ventilation duration** | | | | | | | | | | | | |
| 5^g^ | observational studies | serious^h^ | not serious^i^ | not serious^c^ | serious^j^ | none | 1478 | 290 | - | MD **2.12 lower** (3.86 lower to 0.38 lower) | ⨁◯◯◯ Very low | IMPORTANT |
| **Ventilator Free Days** | | | | | | | | | | | | |
| 4 | observational studies | not serious | very serious^k^ | not serious^c^ | very serious^l^ | none | 291 | 224 | - | MD **0.84 lower** (4.8 lower to 3.12 higher) | ⨁◯◯◯ Very low | IMPORTANT |
| **Continuous Renal Replacement Therapy** | | | | | | | | | | | | |
| 4 | observational studies | not serious | very serious^m^ | not serious^c^ | not serious^n^ | none | 42/213 (19.7%) | 38/168 (22.6%) | **RR 0.65** (0.27 to 1.55) | **79 fewer per 1,000** (from 165 fewer to 124 more) | ⨁◯◯◯ Very low | IMPORTANT |

**CI:** confidence interval; **MD:** mean difference; **RR:** risk ratio

#### Explanations

a. Studies included are based on low to moderate risk of bias

b. Moderate heterogeneity is detected (I2= 43%) [Supplementary Fig. S64]

c. No serious indirectness related to PICO elements and methodological elements

d. We did not downgrade the level of evidence because the pooled effect is between the minimum benefit threshold and null effect showing that early intubation has a trivial effect in reducing mortality [Supplementary Fig. S64]

e. Substantial heterogeneity is detected (I2 = 70%) [Suppelementary Fig. S65]

f. We downgraded one level of evidence because the pooled effect crossed the minimum benefit threshold but the upper confidence interval reach the area of trivial effect. Thus, early intubation probably reduces ICU length of stay [Supplementary Fig. S65]

g. Studies included are based on point of care with low COVID-19 disease burden.

h. Studies included in this outcome has moderate to serious risk of bias

i. No heterogeneity detected (I2 = 0%) [Supplementary Fig. S66]

j. We downgraded one level of evidence because the pooled effect crossed the minimum benefit threshold but the upper confidence interval fall on the area of trivial effect. Thus, early intubation probably reduces ventilation duration [Supplementary Fig. S66]

k. Considerable heterogeneity is detected (I2=75%) [Supplementary Fig. S67]

l. We downgraded two level of evidence because the point estimate of pooled effect is on the area of trivial effect while the confidence interval crossed the minimum benefit and harm threshold. Thus, early intubation may have trivial or no effect in VFD [Supplementary Fig. S67]

m. Considerable heterogeneity is detected (I2=89%) [Supplementary Fig. S68]

n. We did not downgraded the level of evidence because the pooled effect is between the minimum benefit and harm threshold. Thus, early intubation has a trivial or no effect for risk in using CRRT [Supplementary Fig. S68]
