# Supplementary material for: Outcome in early vs late intubation among COVID-19 patients with acute respiratory distress syndrome: an updated systematic review and meta-analysis
Source: Sci Rep. 2022 Dec 14;12:21588. doi: 10.1038/s41598-022-26234-7 (PMC9748395; doi:10.1038/s41598-022-26234-7)
Supplement: Supplementary file 5 — Supplementary Information 5. [file 41598_2022_26234_MOESM5_ESM.docx]

**Supplementary Table S4**

**Manuscript Title:** Outcome in Early vs Late Intubation among COVID-19 Patients with Acute Respiratory Distress Syndrome: An Updated Systematic Review and Meta-Analysis

**Author List:** Denio A. Ridjab^1^, Ignatius Ivan^2^, Fanny Budiman^2^, Dafsah A. Juzar^3^

^1^Department of Medical Education Unit, School of Medicine and Health Sciences, Atma Jaya Catholic University of Indonesia, Jakarta, Indonesia

^2^School of Medicine and Health Sciences, Atma Jaya Catholic University of Indonesia, Jakarta, Indonesia

^3^Department of Cardiology and Vascular Medicine, Faculty of Medicine, Universitas Indonesia/Harapan Kita National Cardiovascular Centre

**Supplementary Table S4.** Characteristic of Included Studies

| **Author (Year)** | **Study Design** | **Country** | **Subjects Total (% male)** | **Ages, Years^a^** | **Follow Up^a^** | **Inclusion and Exclusion Criteria** | **Clinical Course (EI vs LI)^a^** | **Comorbidity** | **Definition of Intubation Timing** | **ICU-Mortality Total (%)** | **Secondary Outcome** |
| --- | --- | --- | --- | --- | --- | --- | --- | --- | --- | --- | --- |
| Bavishi, et al. (2021) | Single-center, retrospective cohort | United States | All patients:  54 (68.5%)  EI (4-24 hours): 30 (70%)  LI (5-10 days): 24 (66%)  Prior HFNC or NIV trial: 36 | EI: 58 [42–69]  LI: 62 [50–69]  p=0.32 | EI: 19 days [10–28]  LI: 29 days [19–33] | Inclusion: Hospitalized adults (>18 years old) with RT-PCR-confirmed SARS-CoV-2 infection and having ARDS in accordance with Berlin criteria  Exclusion: Patients intubated  within 4 hours of arrival | PF Ratio: 184 [114-245] vs 186 [116-235], p=0.93  SOFA ICU: 6 [3-8] vs 4 [1-7], p=0.33 | EI vs LI:  Hypertension: 13 (43%) vs 18 (75%), p<0.01  DM: 13 (43%) vs 13 (54%), p=0.28  CKD: 3 (10%) vs 9 (37.5%), p<0.001  ESRD: 1 (3%) vs 6 (25%), p<0.001  CAD: 4 (13%) vs 3 (12.5%), p=0.9  CHF: 4 (13%) vs 2 (8%), p=0.47  COPD: 4 (13%) vs 2 (8%), p=0.47  Asthma: 1 (3%) vs 1 (4%) | EI: patients intubated between 4 and 24 hours of hospital admission  LI: patients intubated between 5 and 10 days of hospital admission | EI: 2 (6%)  LI: 7 (29%)  P<0.001 | ICU LOS: 12 [5–17] vs 15 [10–19] [Mean, SD: 11.3 (9.3) vs 14.7 (7.1)]*, p>0.05  Ventilation duration: 10 (5-15) vs 10 (7-19) [Mean, SD: 10 (7.8) vs 12 (9.5)]*, p>0.05  Adverse events:  CRRT: 3 (10%) vs 7 (29%), p=0.07 |
| COVID-ICU Group (2021) | Multicenter, prospective cohort | France, Switzerland, Belgium | All patients: 4244 (74%)  EI (≤ 24 hours): 2635  LI (> 24 hours): 741  Prior HFNC or NIV trial: N.R | All patients:  63.0 [54.0–71.0] | 90 days | Inclusion: Hospitalized adults (>18 years old) with RT-PCR-confirmed SARS-CoV-2 infection and having ARDS in accordance with Berlin criteria | All Patients:  PF Ratio: 154 [106–223]  SOFA ICU: 5.0 [3.0–8.0]  SAPS II: 37 [28–50] | All Patients:  Hypertension = 2018 (48%)  DM = 1167 (28%)  Immunodeficiency = 314 (7%)  Obesity (BMI > 30 Kg/m^2^) = 1607 (41%) | EI: patients intubated within 24 hours of ICU admission  LI: patients intubated later than 24 hours of ICU admission | EI: 957 (36.3%)  LI: 244 (32.9%)  p>0.05 | N.R |
| Dupuis, et al. (2021) | Multicenter, prospective Cohort | France | All patients:  245 (76.4%)  EI (≤48 hours): 117 (80.3%)  Mild AHRF: 9  Moderate AHRF: 61  Severe AHRF: 47  LI (>48 hours): 45 (66.7%)  Mild AHRF: 10  Moderate AHRF: 21  Severe AHRF: 14  Prior HFNC or NIV trial: 146 | EI: 61 [52-69]  LI: 63 [53-70]  p>0.05 | 60 days | Inclusion: Hospitalized adults (>18 years old) with AHRF related to severe COVID-19 pneumonia defined as the combination of: 1) radiological features compatible with this diagnosis, 2) Pao2/Fio2 ratio ≤ 300 mm Hg, and 3) a positive SARS-CoV-2 test using RT-PCR  Exclusion: referral from another ICU or intermediate care unit, when a decision was made to discontinue life-sustaining treatments during the first 2 days after ICU admission, if ICU LOS was ≤ 2 days and if they had a shock or a GCS ≤ 12 on ICU admission | RR: 28 (24- 33) vs 32 (29-40), p<0.05  PF Ratio:  110 [80-155] vs  123.33 [90-194.46], p>0.05  SAPS II score:  39 [31-51] vs  34 [27-39], p<0.05 | EI vs LI:  Liver disease: 1 (0.85%) vs 1 (2.22%), p=0.48**  Cardiovascular disease: 33 (28.21%) vs 8 (17.78%), p=0.17**  Respiratory disease: 12 (10.26%) vs 8 (17.78%), p=0.19**  CKD: 10 (8.55%) vs 6 (13.33%), p=0.36**  Immunosuppression: 8 (6.84%) vs 7 (15.56%), p=0.09** | EI: patients intubated within 48 hours of ICU admission  LI: patients who received at least one of these NIV: NIPPV, HFNC, CPAP, and NRM and not earlier than the third day after ICU admission. | EI: 48 (41.03%)  LI: 18 (40%)  p=0.91* | ICU LOS: 15 [10-21] vs 16 [11-22] [Mean, SD: 15.3 (8.3) vs 16.3 (8.4)]*, p=0.49**  VFD: 1 [0-3] vs 4 [2-6] [Mean, SD: 1.3 (2.3) vs 4 (3.1)]*, p<0.01** |
| Fayed, et al. (2021) | Single-center, retrospective cohort | United States | All patients:  110  EI (≤ 24 hours): 38  LI (> 24 hours): 51  Prior HFNC or NIV trial: 58 | EI: 62.6 (12.8)  LI: 63.4 (15.9)  p=0.40 | 18.5 days [13-29] | Inclusion: Hospitalized adults (>18 years old) with RT-PCR-confirmed SARS-CoV-2 infection with severe respiratory distress syndrome and with bilateral infiltrates indicated by chest X-ray in addition to at least one of the following criteria: RR > 30 for at least 2 hours and SaO2 <93% for at least 2 hours.  Exclusion: “do not intubate” status | SOFA ICU: 5.9 [5.7-6.6] vs 6.3 [5.8-6.7], p=0.14  ROX index:  Low intubation risk: 18 (47%) vs 15 (29%)  Intermediate intubation risk:  1 (3%) vs 8 (16%)  High intubation risk:  19 (50%) vs 28 (55%) | EI vs LI:  Acute renal failure: 22 (57.9%) vs 37 (51.4%), p=0.15  Dialysis: 9 (24%) vs 15 (29%), p=0.5 | EI: patients intubated within 24 hours of ARDS onset meeting inclusion criteria  LI: patients intubated later than 24 hours of ARDS onset meeting inclusion criteria | EI: 10 (26.3%)  LI: 25 (49%)  p=0.03 | ICU LOS: 19 [13-28] vs 17 [11-28] [Mean, SD: 20 (11.5) vs 18.7 (13)]*, p=0.438  Ventilation duration: 17 [10-25] vs 10 [5-22] [Mean, SD: 17.3 (11.6) vs 12.3 (13)]*, p=0.055 |
| Ferraz, et al. (2021) | Single-center, retrospective cohort | Portugal | All patients:  177 (68%)  EI (≤ 24 hours): 88 (72%)  LI (> 24 hours): 89 (66%)  Prior HFNC or NIV trial: 89 | EI: 64  LI: 63  p=0.6 | 21 days | Inclusion: Hospitalized adults (>18 years old) with RT-PCR-confirmed SARS-CoV-2 infection and having ARDS in accordance with Berlin criteria | All patients:  PF Ratio: < 150 | N.R | EI: Patients  intubated within 24-h of a PF ratio lower than 150 without NIV or HFNC  LI: patients intubated with a P/F ratio lower than 150 after a 24 h trial of NIV, HFNC or a combination of these two modalities | EI: 24 (88)  LI: 27 (89)  p=0.64 | ICU LOS: 11 days vs 10 days, p=0.46  Ventilation duration: 9 days vs 9 days, p=0.68  Adverse events:  VAP: 31 (35.4%) vs 46 (51.4%), p=0.036 |
| Grasselli, et al. (2020) | Multicenter, retrospective cohort | Italy | All patients:  3988 (80%)  EI (≤ 24 hours): 2929  LI (> 24 hours): 164  Prior HFNC or NIV trial: N.R | All patients:  63 [56-69] | 70 days [61-70] | Inclusion: Hospitalized adults (>18 years old) with RT-PCR-confirmed SARS-CoV-2 infection and having ARDS in accordance with Berlin criteria | All patients:  PF Ratio  <103: 729 patients  103-144: 728 patients  145-203: 729 patients  >203: 728 patients | All patients:  Hypertension = 1643 (41.2%)  Hypercholesterolemia = 545 (13.7%)  Heart disease = 533 (13.4%)  DM = 514 (12.9%)  Malignancy= 331 (8.3%)  COPD = 93 (2.3%)  CKD = 87 (2.2%)  Liver disease = 86 (2.2%) | EI: patients intubated within 24 hours of ICU admission  LI: patients intubated later than 24 hours of ICU admission | EI: 1514 (51.7%)  LI: 79 (48.2%)  p>0.05 | N.R |
| Hernandez-Romieu, et al. (2020) | Multicenter, retrospective cohort | United States | All patients:  175 (55.4%)  EI:  <8 hour = 76 (50%)  8-24 hour = 57, (59.6%)  LI (>24 hours): 42 (59.5%)  Prior HFNC or NIV trial: 78 | EI:  <8 hour = 67 [56–76]  8-24 hour = 65 [55–73]  LI: 67 [57–77]  p=0.6 | 12.8 days [7.5-17.8] | Inclusion: Hospitalized adults (>18 years old) with RT-PCR-confirmed SARS-CoV-2 infection and having ARDS in accordance with Berlin criteria  Exclusion: intubation prior to transfer from another facility, “do-not-intubate” status, less than 12-h of ICU admission | PF Ratio:  EI:  <8 hour = 163 [110–214]  8-24 hour = 136 [110–182]  LI: 150 [115–192]  p=0.7  SOFA ICU:  EI:  <8 hour = 10.5 [9.0–12.5]  8-24 hour = 9.0 [7.0–12.0]  LI: 7.5 [6.0–9.0]  p< 0.0001 | Hypertension =  EI:  <8 hour = 33 (43.4%)  8-24 hour = 26 (45.6%)  LI: 18 (42.9%)  p=0.09  DM =  EI:  <8 hour = 53 (69.7%)  8-24 hour = 29 (50.9%)  LI: 18 (42.9%)  p=0.006  CKD =  EI:  <8 hour = 27 (35.5%)  8-24 hour = 13 (22.8%)  LI: 10 (23.8%)  p=0.2  ESRD =  EI:  <8 hour = 11 (14.5%)  8-24 hour = 10 (17.5%)  LI: 1 (2.4%)  p=0.04  COPD =  EI:  <8 hour = 14 (18.4%)  8-24 hour = 10 (17.5%)  LI: 9 (21.4%)  p=0.9 | EI: patients intubated within 8 hours and 8-24 hours of ICU admission  LI: patients intubated later than 24 hours of ICU admission | EI:  <8 hour: 29 (38.2%)  8-24 hour: 18 (31.6%)  LI: 16 (38.1%)  p=0.7  OR^c^:  8-24 hour vs <8 hour: 0.73 (0.29–1.85)  ≥ 24 hour vs <8 hour: 2.34 (0.73–7.44) | ICU LOS:  EI (≤ 24 hours): 12.56 (7.87)  LI (> 24 hours): 13 (8.1)  p>0.05  Ventilator duration:  EI (≤ 24 hours): 10.05 (7.99)  LI (> 24 hours): 9 (6.14)  p>0.05 |
| Hyman, et al. (2020) | Multicenter, retrospective cohort | United States | All patients:  755 (64%)  EI (≤ 48 hours): 313  LI (> 48 hours): 442  Prior HFNC or NIV trial: 72 | All patients:  63 (13) | 4 months | Inclusion: Hospitalized adults (>18 years old) with RT-PCR-confirmed SARS-CoV-2 infection and having ARDS in accordance with Berlin criteria | N.R | All patients:  Asthma/COPD = 47 (6.3%)  Cancer = 47 (6.3%)  CHF = 30 (4.3%)  CKD = 69 (9.2%)  DM = 177 (23.7%)  Hypertension = 248 (33.1%)  Liver disease = 10 (1.4%)  Stroke = 37 (4.9%) | EI: patients intubated within 48 hours of hospital admission  LI: patients intubated later than 48 hours of hospital admission | EI: 199 (63.6%)  LI: 313 (70.8%)  P<0.05 | N.R |
| Karagiannidis, et al. (2020) | Multicenter, retrospective cohort | Germany | All patients:  1727 (66.4%)  EI (≤ 24 hours): 1318 (66.5%)  LI (> 24 hours): 141 (70.2%)  Prior HFNC or NIV trial: 141 | EI: 67.9 (13.1)  LI: 67.6 (12.9)  p>0.05 | 90 days | Inclusion: Hospitalized adults (>18 years old) with RT-PCR-confirmed SARS-CoV-2 infection and having ARDS in accordance with Berlin criteria | N.R | EI vs LI:  Hypertension: 816 (61.9%) vs 91 (64.5%), p>0.05  DM: 520 (39.5%) vs 60 (42.6%), p>0.05  Cardiac arrhythmia: 574 (43.6%) vs 64 (45.4%), p>0.05  Renal failure: 306 (23.2%) vs 28 (19.9%)  CHF: 385 (29.2%) vs 45 (31.9%)  COPD: 234 (17.8%) vs 29 (20.6%)  Obesity: 182 (13.8%) vs 23 (16.3%)  Dialysis: 404 (30.7%) vs 41 (29.1%), p>0.05 | EI: intubation within 24-h of ICU admission or intubation without NIV  LI: NIV failure and intubated after 24-h of ICU admission | EI: 696 (52.8%)  LI: 70 (49.6%)  p>0.05 | ICU-LOS: 26.5 (18.7) vs 29.9 (19.7), p>0.05  Ventilation duration: 15.1 (12.1) vs 17.1 (12.7), p>0.05 |
| Lee, et al. (2020) | Multicenter, retrospective cohort | South Korea | All patients:  47 (59.6%)  EI (≤ 24 hours): 23 (60.9%)  LI (>24 hours)I: 16 (62.5%)  Prior HFNC or NIV trial: 29 | EI: 72 [64-76]  LI: 66 [59-77]  p>0.05 | 46 days [24-86] | Inclusion: Hospitalized adults (>18 years old) with RT-PCR-confirmed SARS-CoV-2 infection and having ARDS in accordance with Berlin criteria  Exclusion: “do not intubate” status | MAP: 93 [90–107] vs 93 [86–97], p=0.388  RR: 28 [22–34] vs 21 [20–29], p= 0.057  PF Ratio: 86 [69–123] vs 120 [62–188], p=0.204  SOFA ICU: 3 [2–7] vs 3 [2–4], p=0.336  APACHE II Score: 15 [10–17] vs 14 [8–15], p=0.252 | EI vs LI:  Hypertension: 10 (43.5%) vs 8 (50%), p=0.688  DM: 10 (43.5%) vs 7 (43.8%), p=0.987  CKD: 1 (4.3%) vs 2 (12.5%), p=0.557  Dementia: 2 (8.7%) vs 1 (6.2%), p>0.999  Cerebrovascular disease: 0 (0%) vs 1 (6.2%), p=0.410  Malignancy: 2 (8.7%) vs 4 (25%), p=0.205  Cardiovascular disease: 4 (17.4%) vs 2 (12.5%), p>0.999  COPD: 3 (13%) vs 1 (6.2%), p=0.631  Chronic liver disease: 1 (4.3%) vs 0 (0%), p>0.999 | EI: patients intubated within 24 hours of ARDS onset  LI: patients intubated later than 24 hours of ARDS onset | EI: 13 (56.5%)  LI: 7 (43.8%)  p=0.433  HR^d^ for EI vs LI:  1.964, (0.351–11.004), p=0.442 | ICU LOS: 13 [7–33] vs 47 [13–74] [Mean, SD: 17.7 (19.5) vs 44.7 (49.6)]*, p=0.101  Ventilator duration: 12.67 (15.8) vs 28.67 (39.02), p>0.05  VFD: 9 [0–18] vs 25 [7–45] [Mean, SD: 9 (14.2) vs 25.7 (30.9)]*, p=0.033  Adverse events:  Septic shock: 20 (87%) vs 14 (87.5%), p>0.999  Acute kidney injury: 10 (43.5%) vs 7 (43.8%), p=0.987  Acute cardiac injury: 10 (43.5%) vs 4 (25%), p=0.237  VAP or HAP: 7 (30.4%) vs 1 (6.2%), p=0.109  CRBSI: 4 (17.4%) vs 3 (18.8%), p>0.999  Bleeding: 3 (13%) vs 3 (18.8%), p=0.674  CPCR: 1 (4.3%) vs 2 (12.5%), p=0.557 |
| Matta, et al. (2020) | Single-center, retrospective cohort | United States | All patients:  111 (54.1%)  EI (≤ 48 hours): 76 (55%)  LI (> 48 hours): 35 (51%)  Prior HFNC or NIV trial: 81 | EI: 69.79 (12.15)  LI: 65.03 (8.37)  p=0.038 | EI: 13.98 days (8.71)  LI: 15.15 days (8.11) | Inclusion: Hospitalized adults (>18 years old) with RT-PCR-confirmed SARS-CoV-2 infection and having ARDS in accordance with Berlin criteria | PF Ratio: 106 (63–169) vs 111 (60–178), p=0.644  SOFA ICU: 8.15 (3.29) vs 6.29 (2.80), p=0.005 | EI vs LI:  COPD: 10 (13%) vs 6 (17%), p>0.05  Asthma: 5 (7%) vs 4 (11%), p>0.05  Sleep apnea: 10 (13%) vs 3 (9%), p>0.05  CHF: 15 (20%) vs 9 (26%), p>0.05  Atrial fibrillation: 12 (16%) vs 2 (6%), p>0.05  Liver cirrhosis: 2 (3%) vs 3 (9%), p>0.05  DM: 45 (59%) vs 18 (51%), p>0.05  CKD: 14 (18%) vs 7 (20%), p>0.05  HIV: 1 (1%) vs 0 (0%), p>0.05  CAD: 18 (24%) vs 10 (29%), p>0.05  Hypertension: 66 (87%) vs 31 (89%), p>0.05 | EI: intubation at admission or within 48-h since the onset of increased oxygen requirements (require >50% of Fio2 which is requiring >10 L of nasal cannula, or NRM, or HFNC, or NIPPV)  LI: intubation later than 48-h since the onset of increased oxygen requirement | EI: 54 (71%)  LI: 20 (57%)  p=0.194 | ICU-LOS: 10.76 (7.59) vs 8.19 (7.53), p=0.103  Ventilator duration: 10.41 (7.53) vs 8.00 (7.82), p=0.125  Adverse events:  CRRT: 23 (30%) vs 7 (20%), p=0.358 |
| Mellado-Artigas, et al. (2021) | Multicenter, prospective cohort | Spain  Andorra | All patients:  468 (69.2%)  EI (≤ 24 hours): 312 (67.6%)  LI (> 24 hours): 49  Prior HFNC or NIV trial: 92 | EI: 62.3 (10.9)  LI: 60.6 (12.7)  p=0.10 | 60 days | Inclusion: Adult patients (18 years or older) with COVID-19 related acute respiratory failure suitable for either a conservative or early intubation strategy; PaO2/FiO2 ratio < 300 mmHg; RR on day 1 <35 breaths/min; GCS <13 and pH ≤7.25 | RR: 24.7 (6) vs 25.3 (5.2), p=0.26  PF ratio: 121.2 (58) vs 122.7 (48.5), p=0.78  SOFA ICU: 7 [5-8] vs 4 [3-5], p<0.01  ROX Index: 4 [3.2-6] vs 5.1 [4.1-6.6], p<0.01 | N.R | EI: patients intubated within 24 hours of ICU admission  LI: patients intubated later than 24 hours of ICU admission | EI: 99 (31.7%)  LI: 15 (30.6%)  p>0.05 | N.R |
| Pandya, et al. (2021) | Single-center, retrospective cohort | United States | All patients:  75 (57.3%)  EI (≤1.27 days): 37 (57.3%)  LI (>1.27 days): 38 (48.8%)  Prior HFNC or NIV trial: N.R | EI: 65.92 (14.79)  LI: 64.05 (13.87)  p=0.58 | 1 month | Inclusion: Hospitalized adults (>18 years old) with RT-PCR-confirmed SARS-CoV-2 infection and having ARDS in accordance with Berlin criteria | PF Ratio: 205.5 (106 - 378) vs 160.00 (99-  268), p=0.46 | N.R | EI: patients intubated within 1.27 days of ICU admission  LI: patients intubated later than 1.27 days of ICU admssion | EI: 17 (45.95%)  LI: 20 (54.05%)  p=0.563 | ICU LOS: 7.38 [3.88-10.21] vs 12.31 [7.75-19.96] [Mean, SD: 7.16 (4.88) vs 13.34 (9.41)]*, p=0.001  Ventilator duration: 5.86 (8.40) vs 10.30 (8.78), p=0.102 |
| Parish, et al. (2021) | Multicenter, retrospective cohort | United States | All patients:  1628  EI: 807  LI: 821  Prior HFNC or NIV trial: 399 | EI vs LI >65 years = 361 (44.7%) vs 359 (44.5%), p=0.92 | 60 days | Inclusion: Hospitalized adults (>18 years old) with RT-PCR-confirmed SARS-CoV-2 infection and having ARDS in accordance with Berlin criteria | N.R | EI vs LI:  Asthma = 115 (14.3%) vs 111 (13.8%), p=0.77  COPD = 79 (9.8%) vs 71 (8.8%), p=0.49  CAD = 99 (12.3%) vs 101 (12.5%), p=0.98  CHF = 91 (11.3%) vs 92 (11.4%), p=0.99  CKD = 65 (8.1%) vs 66 (8.2%), p=0.98  ESRD = 43 (5.3%) vs 46 (5.7%), p=0.74  DM = 397 (49.2%) vs 415 (51.4%)  Hypertension = 527 (65.3%) vs 546 (67.7%)  Cancer = 49 (6.1%) vs 62 (7.7%) | EI: patients intubated within 48 hours of ICU admission  LI: patients intubated later than 48 hours of ICU admission | EI: 254 (31.5%)  LI: 237 (28.9%)  p>0.05  HR^e^ for EI vs LI: 1.09, [0.94–1.26]  p = 0.26 | N.R |
| Roedl, et al. (2021) | Multicenter, retropective cohort | Germany | All patients:  223 (73%)  EI (≤ 24 hours): 128  LI (> 24 hours): 39  Prior HFNC or NIV trial: 46 | All patients:  69.0 [58.0–77.5] | 13 days [5-24] | Inclusion: Hospitalized adults (>18 years old) with RT-PCR-confirmed SARS-CoV-2 infection and having ARDS in accordance with Berlin criteria  Exclusion: Patients with noncompleted ICU stay (ongoing ICU treatment) | All patients:  SOFA ICU: 5.0 [3.0–9.0] | All patients:  Hypertension = 108 (48.4%)  Obesity (BMI>30Kg/m^2^) = 52 (23.3%)  DM = 59 (26.5%)  Immunosuppresion = 38 (17%)  CAD = 18 (8.1%)  CHF = 15 (6.7%)  Connective tissue disease = 6 (2.7%)  CKD = 26 (11.7%)  COPD = 40 (17.9%)  Chronic liver disease = 5 (2.2%)  Lymphoma = 12 (5.4%)  Leukaemia = 17 (7.6%)  Solid organ tumor = 15 (6.7%) | EI: patients intubated within 24 hours of ICU admission  LI: patients intubated later than 24 hours of ICU admission | EI: 55 (43%)  LI: 19 (48.7%)  p>0.05 | N.R |
| Saida, et al. (2021) | Single-center, retrospectivecohort | Tunisia | All patients:  10 (80%)  EI (≤ 24 hours): 4  LI (> 24 hours): 3  Prior HFNC or NIV trial: 1 | All patients:  51.8 (6.3) | 11.2 days (5.8) | Inclusion: Hospitalized adults (>18 years old) with RT-PCR-confirmed SARS-CoV-2 infection and having ARDS in accordance with Berlin criteria | All patients:  PF Ratio: 136.2 (79.7) | All patients:  Hypertension = 1 (10%)  Diabetes = 6 (60%)  Obesity = 2 (20%)  Asthma = 1 (10%)  CKD = 2 (20%) | EI: patients intubated within 24 hours of ICU admission  LI: patients intubated later than 24 hours of ICU admission | EI: 4 (100%)  LI: 3 (100%)  p>0.05 | N.R |
| Siempos, et al. (2020) | Single-center, retrospective cohort | Greece | EI (≤ 24 hours): 19 (57%)  LI (> 24 hours): 14 (89%)  Prior HFNC or NIV trial: 11 | EI: 63 [57-69]  LI: 68 [58-75]  p=0.37 | 28 days | Inclusion: Hospitalized adults (>18 years old) with RT-PCR-confirmed SARS-CoV-2 infection and with AHRF defined as the requirement of > 5 L/min nasal oxygen (or Venturi mask more than 40%) to keep a pulse oximeter measured arterial blood oxygen saturation (SpO2) ≥ 95%.  Exclusion: “do not intubate” status | SOFA ICU: 4 [4-5] vs 5 [4-6], p=0.4 | EI vs LI:  Cardiovascular disease: 7 (50%) vs 9 (50%), p=1  DM: 2 (14%) vs 4 (22%), p=0.67  COPD: 1 (7%) vs 1 (6%), p=1  ESRD: 0 (0%) vs 1 (6%), p=1  Malignancy: 0 (0%) vs 4 (22%), p=0.11 | EI: patients intubated within 24 hours of ICU admission  LI: patients receiving NRM for later than 24-h or HFNC for any period of time or NIV for any period of time in an attempt to avoid intubation | EI: 6 (31.6%)  LI: 5 (35.7%)  p>0.05 | VFD: 3 [0-17] vs 2 [1-13] [Mean, SD: 6.7 (14) vs 5.3 (9.7)]*, p=0.57  Ventilator duration: 15.89 (10.27) vs 14.29 (7.99), p>0.05  ICU-free days: 0 [0-16] vs 0 [0-4] [Mean, SD: 5.3 (13.2) vs 1.3 (3.2)]*, p=0.39  Adverse Events:  Septic shock: 6 (32%) vs 11 (79%), p=0.3  CRRT: 4 (21%) vs 12 (63%), p=0.07 |
| Vera, et al. (2021) | Single-centre, prospective cohort | Chile | All patients:  183 (72%)  EI (≤ 48 hours) : 88 (71%)  LI (> 48 hours): 95 (74%)  Prior HFNC or NIV trial: 244 | EI: 59 [53–66]  LI: 64 [55–71]  p=0.013 | 28 days | Inclusion: Hospitalized adults (>18 years old) with RT-PCR-confirmed SARS-CoV-2 infection and having ARDS in accordance with Berlin criteria | PF Ratio: 123 [82-166] vs 99 [77-158], p=0.18  SOFA ICU: 6 [4-8] vs 4 [2-8], p=0.014  APACHE: 12 [8-15] vs 13 [8-20], p=0.35 | EI vs LI:  DM: 27 (31%) vs 34 (36%), p=0.46  Hypertension: 41 (47%) vs 46 (48%), p=0.69 | EI: patients intubated within 48 hours of hospital admission  LI: patients intubated later than 48 hours of hospital admission | EI: 16 (18%)  LI: 43 (43%)  p<0.001 | ICU LOS: 15 [9–23] vs 23 [12–39] [Mean, SD: 15.7 (10.6) vs 24.7 (20.3)], p=0.003  Ventilation duration: 13 [8–25] vs 16 [9–33] [Mean, SD: 15.3 (12.8) vs 19.3 (18.1)]*, p=0.131  VFD: 15 [3−20] vs 12 [0–19] [Mean, SD: 12.7 (12.8) vs 10.3 (14.3)]*,p=0.196  Adverse Events:  CRRT: 12 (14%) vs 12 (13%), p=0.841 |
| Zirpe, et al. (2021) | Single-center, retrospective cohort | India | All patients:  147  EI: 75 (74.6%)  LI: 72 (73.6%) | EI: 58 [50–69]  LI: 59 [52–67]  p=0.9 |  | Inclusion: Hospitalized adults (>18 years old) with RT-PCR-confirmed SARS-CoV-2 infection and having ARDS in accordance with Berlin criteria | PF Ratio: 74.7 [60.0–104.7] vs 70.7 [57.3–82.4], p=0.2  qSOFA ICU: 1 [1-2] vs 1 [1-2], p=0.7  APACHE II: 12 [9-15] vs 10 [8-13], p=0.03 | EI vs LI:  DM: 35 (46.6%) vs 31 (43.1%), p=0.6  Hypertension: 35 (46.6%) vs 29 (40.3%), p=0.4  CKD: 5 (6.6%) vs 3 (4.2%), p=0.5  CAD: 9 (12%) vs 10 (13.9%), p=0.7  COPD: 1 (1.3%) vs 0 (0%), p=0.7  Obesity: 1 (1.3%) vs 2 (2.8%), p=0.5 | EI: patients intubated within 48 hours of ICU admission  LI: patients intubated later than 48 hours of ICU admission | EI: 45 (60%)  LI: 56 (77.7%)  p=0.02 | ICU-LOS: 14 (9.7–21) vs 16 (7–21.7) [Mean, SD: 14.9 (8.5) vs 14.9 (11.1)], p=0.9  Ventilation duration: 7 [4–12] vs 6 [2–12] [Mean, SD: 7.7 (6.0) vs 6.7 (7.6)]*. p=0.2 |
| Zuccon, et al. (2021) | Single-center, retrospective cohort | Italy | All patients:  54 (81.5%)  EI (≤ 24 hours): 25  LI (> 24 hours): 23  Prior HFNC or NIV trial: 23 | All patients:  30-39 years: 1  40-49 years: 7  50-59 years: 11  60-69 years: 20  70-79 years: 14  80-89 years: 1 | 56 days | Inclusion: Hospitalized adults (>18 years old) with RT-PCR-confirmed SARS-CoV-2 infection and having ARDS in accordance with Berlin criteria | N.R | N.R | EI: patients intubated within 24 hours of ICU admission  LI: patients intubated later than 24 hours of ICU admission | EI: 9 (36%)  LI: 12 (52.2%)  p>0.05 | N.R |

AHRF, acute hypoxemic respiratory failure; APACHE, Acute Physiology and Chronic Health Evaluation; ARDS, acute respiratory distress syndrome; CAD: coronary artery disease; CHF: congestive heart failure; CKD: chronic kidney disease; COPD, chronic obstructive pulmonary disease; CPAP, continuous positive airway pressure; CPCR, cardiopulmonary–cerebral resuscitation; CRBSI, catheter–related bloodstream infection; DM, diabetes mellitus; EI, early intubation; ESRD, End-Stage Renal Disease; GCS, Glasgow Coma Scale; HAP, hospital–acquired pneumonia; HFNC, high-flow nasal cannula; HIV: Human Immunodeficiency Virus; ICU, intensive-care unit; IQR, interquartile range;LI, late intubation; LOS, length of stay; MAP, mean arterial pressure (mmHg); MV, mechanical ventilator; N.R, not reported; NIPPV, non-invasive positive pressure ventilation; NIV, non-invasive ventilation; NRM, non-rebreather mask; p, p value; PF ratio, P_a_O2:F_I_O2 ratio; RR, respiratory rate (breaths/minute); CRRT, continuous renal replacement therapy; RT-PCR, reverse-transcriptase polymerase chain reaction; SARS-CoV-2, severe acute respiratory syndrome coronavirus 2; SD, standard deviation; SOFA, Sequential Organ Failure Assessment; SAPS II, Simplified Acute Physiology Score II; VAP, ventilator–associated pneumonia; VFD, ventilator-free days

^a^Data on age and follow up and clinical course are presented as means (SD) or medians [IQR], as provided by the individual studies

^b^Adjusted for age, body mass index, sex, risk factors and confounders

^c^Adjusted for age, body mass index, sex, race, Elixhauser index, Sequential Organ Failure Assessment score at admission to ICU, time from hospital to ICU admission, and exposure to high-flow nasal cannula

^d^Adjusted for respiratory rate, arterial pH, P_a_CO_2_, P_a_O_2_/F_I_O_2_ ratio, use of high flow nasal cannula, plateau pressure, Ventilator-Associated Pneumonia during ICU stay, and Acute Physiologic Assessment and Chronic Health Evaluation II score

^e^Adjusted for age, sex, race, ethnicity, hospital location, smoking status, and comorbidities

* Mean and standard deviation was estimated from median and interquartile range using Wan’s Method (Wan et al., 2014)

**p-value was estimated using MedCalc Statistical Software version 19.2. 6 (MedCalc Software bv, Ostend, Belgium)
